# Status and Opportunities of Zinc Ion Hybrid Capacitors: Focus on Carbon Materials, Current Collectors, and Separators

**DOI:** 10.1007/s40820-023-01065-x

**Published:** 2023-03-29

**Authors:** Yanyan Wang, Shirong Sun, Xiaoliang Wu, Hanfeng Liang, Wenli Zhang

**Affiliations:** 1 College of Chemistry, Chemical Engineering and Resource Utilization, Northeast Forestry University, 26 Hexing Road, Harbin, 150040 People’s Republic of China; 2 Guangdong Provincial Key Laboratory of Plant Resources Biorefinery, School of Chemical Engineering and Light Industry, Guangdong University of Technology (GDUT), 100 Waihuan Xi Road, Panyu District, Guangzhou, 510006 People’s Republic of China; 3 State Key Laboratory of Physical Chemistry of Solid Surfaces, College of Chemistry and Chemical Engineering, Xiamen University, Xiamen, 361005 People’s Republic of China; 4 Jieyang Branch of Chemistry and Chemical Engineering Guangdong Laboratory (Rongjiang Laboratory), Jieyang, 515200 People’s Republic of China; 5 School of Advanced Manufacturing, Guangdong University of Technology (GDUT), Jieyang, 522000 People’s Republic of China

**Keywords:** Zinc ion hybrid capacitors, Carbon materials, Carbon cathode, Current collectors, Separators

## Abstract

Zinc ion hybrid capacitors (ZIHCs), which integrate the features of the high power of supercapacitors and the high energy of zinc ion batteries, are promising competitors in future electrochemical energy storage applications. Carbon-based materials are deemed the competitive candidates for cathodes of ZIHC due to their cost-effectiveness, high electronic conductivity, chemical inertness, controllable surface states, and tunable pore architectures. In recent years, great research efforts have been devoted to further improving the energy density and cycling stability of ZIHCs. Reasonable modification and optimization of carbon-based materials offer a remedy for these challenges. In this review, the structural design, and electrochemical properties of carbon-based cathode materials with different dimensions, as well as the selection of compatible, robust current collectors and separators for ZIHCs are discussed. The challenges and prospects of ZIHCs are showcased to guide the innovative development of carbon-based cathode materials and the development of novel ZIHCs.

## Highlights

- History and benefits of zinc ion hybrid capacitors are introduced.
- Carbon materials with different dimensions are developed for the cathodes.
- Relationship between carbon structures and capacitive performances are discussed.
- Current collectors and separators are firstly showcased and summarized.

## Graphical abstract

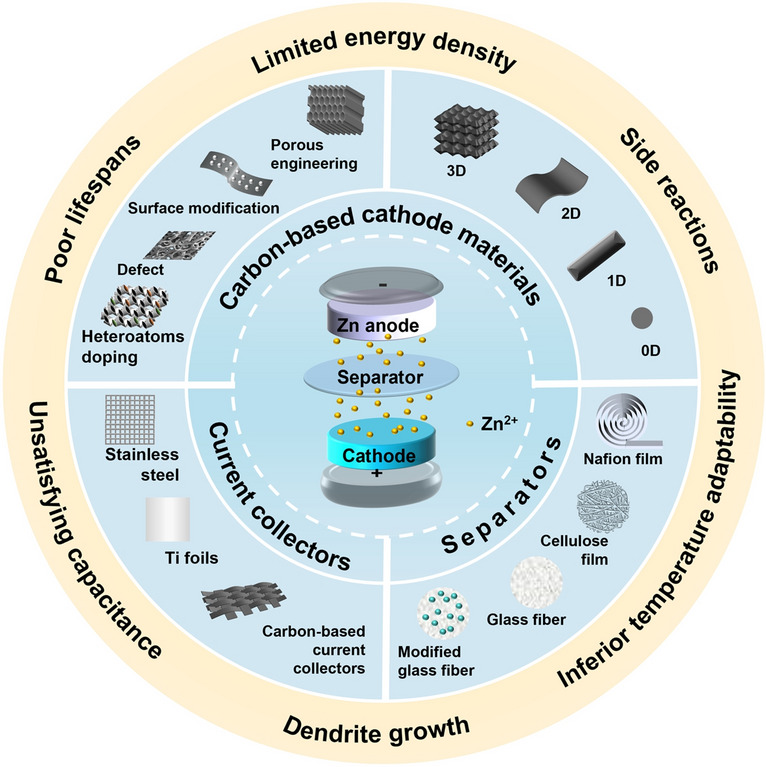

## Introduction

Electrochemical energy storage device is currently regarded as one of the most practical devices for large-scale energy storage of electricity generated by renewable and sustainable energy. According to commercialized electrochemical energy storage devices, relatively mature electrochemical energy storage systems can be commonly categorized as follows: (i) supercapacitors (SCs, also known as electrochemical capacitors) [1, 2], (ii) secondary batteries like lithium-ion batteries, lead-acid batteries, redox flow batteries, sodium-sulfur batteries [3–6], and (iii) fuel cells [7]. SCs have gained tremendous attention due to their long service lifetime, superior power density, wide operating temperature, and high rate capability [8]. For electrochemical energy storage devices, rechargeable batteries, mainly lithium-ion batteries, and lead acid batteries, occupy most of the market. Nevertheless, the major challenges for secondary lithium-ion batteries lie in enormous security concerns resulting from the introduction of flammable organic electrolytes [9], while the challenges for lead-acid batteries are their low energy density and short service life. Hybrid capacitors (HICs), also called asymmetric electrochemical capacitors, are therefore potential energy storage devices that could solve the problems faced by lithium-ion batteries and lead-acid batteries. They are designed to integrate the advantages of SCs and the much higher energy density of rechargeable batteries into one device [10, 11].

**Fig. 1:**
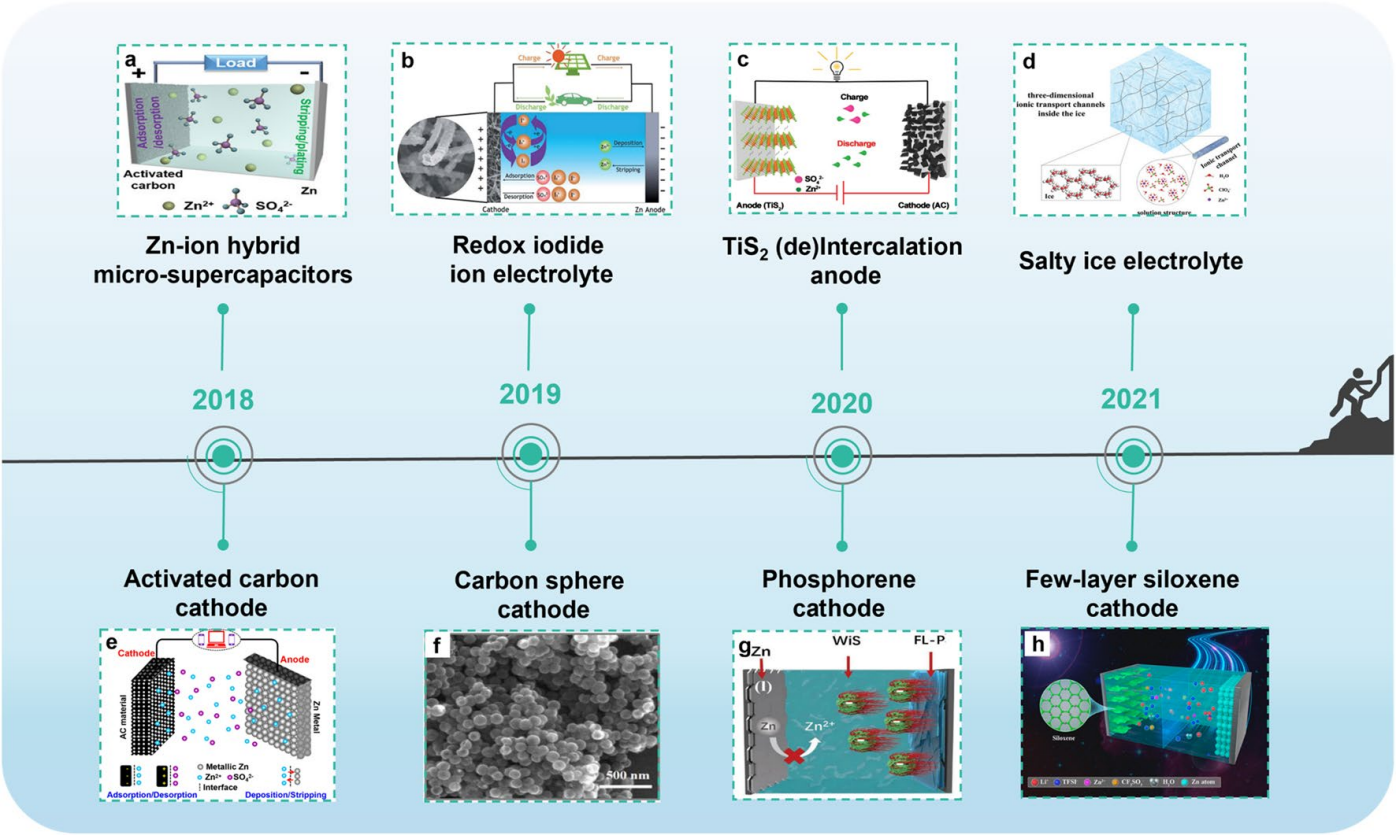
Breakthroughs of ZIHCs in recent years. **a** A new-type Zn-ion hybrid micro-supercapacitor. Reproduced with permission [15]. Copyright 2019, Wiley-VCH. **b** A redox iodide ion electrolyte and B, N dual-doped carbon electrode for ZIHCs. Reproduced with permission [16]. Copyright 2019, Royal Society of Chemistry. **c** Structure and charge storage mechanism of the novel TiS_2_//AC ZIHC. Reproduced with permission [17]. Copyright 2020, Wiley-VCH. **d** Ions transport mechanism in Zn(ClO_4_)_2_ salty ice. Reproduced with permission [31]. Copyright 2021, Wiley-VCH. **e** Activated carbon for cathodes. Reproduced with permission [32]. Copyright 2018, Elsevier. **f** Co-polymer-derived hollow carbon spheres for cathodes. Reproduced with permission [33]. Copyright 2019, Royal Society of Chemistry. **g** Phosphorene as Cathode Material for ZIHCs. Reproduced with permission [19]. Copyright 2020, Wiley-VCH. **h** Few-Layer siloxene as the cathode material for ZIHCs. Reproduced with permission [18]. Copyright 2021, American Chemical Society

Zinc ion hybrid capacitors (ZIHCs) are a tradeoff between zinc ion batteries (ZIBs) and SCs. Although there are many configurations, ZIHCs are mostly composed of a zinc anode, a porous carbon cathode, and Zn^2+^-ion-containing electrolytes [12, 13]. In 2016, Wang et al. constructed the first ZIHC. The ZIHC is comprised of a zinc anode, an oxidized carbon nanotubes (oCNTs) cathode, and ZnSO_4_ aqueous electrolyte [14]. From then on, tremendous investigations on ZIHCs have really been triggered (Fig. 1). For example, the fabrication of zinc ion hybrid micro-supercapacitors filled the blank of multivalent ion-based in-plane micro-supercapacitors for on-chip electronics (Fig. 1a) [15]. The redox iodide ions introduced in ZnSO_4_ electrolytes reduced the charge-transfer resistance (*R*_ct_) and promoted the faradic reactions of the electrode/electrolyte interface for the additional energy storage (Fig. 1b) [16]. Further, titanium disulfide (TiS_2_) was designed as (de)intercalation anode material to replace Zn foil, thus a capacitance retention of 92% was obtained after 5000 cycles (Fig. 1c) [17, 18]. Moreover, new materials that can be used as cathode materials for ZIHCs were explored, such as few-layer phosphorene and few-layer siloxane (Fig. 1g, h) [19]. They are members of few-layer 2D materials family, which have large specific surface area (SSA) and abundant adsorption sites. Based on the different energy storage mechanisms, electrode materials are classified into two types: capacitor-type and battery-type. For the former type, the energy storage mechanism is ions adsorption/desorption on the electrode. Carbon-based materials are the typical capacitor-type electrode materials. They have been found to exhibit tunable porosity, impressive SSA, high electronic conductivity, and good electrochemical stability. Further, due to the merits of low cost, good environmental friendliness and wide application range, carbon materials have been commercialized through mature preparation processes at present [20–22]. Applications of carbon materials in electrochemical devices include active materials, conductive agents, substrates, current collectors, anodic protective layer, active material buffer layers and so on [23–25]. Battery-type electrode materials store and release charges through the deposition/stripping or intercalation/deintercalation of Zn^2+^ ions on the electrode. Metal Zn, manganese-based oxides, and vanadium-based oxides are commonly used battery-type electrode materials in rechargeable zinc ion batteries. The distinct merits of ZIHCs can be described in three aspects. First, the advantages of metal Zn include cost-friendliness, large-scale production, non-toxicity, two-electron redox characteristics, the high gravimetric capacity of 823 mAh g^−1^, and low redox potential (− 0.76 V vs standard hydrogen electrode (SHE)) [26–28]. Compared to lithium and sodium counterparts, ZIHCs based on zinc anode can be assembled in air because of their stability in air and water [29]. Second, using aqueous electrolytes with high ionic conductivity is much safer than employing organic electrolytes. Third, ZIHCs based on the energy storage mechanism of divalent metal ions (Zn^2+^) can provide faster charge transfer kinetics, higher power, and energy density than devices based on the energy storage mechanism of monovalent metal ions [30].

Upgrading ZIHCs toward stable cycling stability and superb energy density has been the primary goal pursued by researchers in recent years. According to the equation of energy density (E) of supercapacitors (Eq. 1),

1 $$ {\text{E}} = 0.5CV^{2} $$

Here, *C* is the capacitance, *V* is the voltage window. Increasing the capacitance of cathode materials and widening the voltage window are effective solutions to enhance the energy densities of ZIHCs. Previous researches have shown that electrode materials store charges through the electric double layer (EDL) capacitance and pseudocapacitance. The EDL capacitance is based on the pure electrostatic attraction between the electrolyte ions and the charged surface of the electrodes. Pseudocapacitance originates from the redox or Faradaic reactions of the electroactive species on the electrode surface. Nano porous carbon materials have recently attracted interest for ZIHC electrodes. However, the overall electrochemical performance of most carbon cathodes reported is still far from practical requirements. By modifying or creating novel carbon materials, the capacitance of carbon-based electrodes can be further enhanced [34]. For instance, the chemical activation process can increase the SSA of carbon materials and form abundant pores, thereby increasing their EDL capacitances [35, 36]. Defect engineering could create the adsorption/desorption sites for electrolyte ions and enhance the rate performance of carbons. Heteroatom doping is commonly used to break cross-linked *sp*^3^ linkage in carbon skeletons and induce heteroatom-induced or carbon-vacancy defects [37]. Introducing pseudocapacitance could also improve the energy storage capability [38]. In addition, as the charge carrier of the electrode, current collectors are one of the indispensable components in ZIHC. Researchers have recently focused on developing lightweight current collectors [39–41]. The weight to volume ratio of lightweight current collectors is lower than conventional metal current collectors, freeing up more space in the device for active materials. With the increase in the portion of active materials in the ZIHC device, the energy density of the devices is expected to reach a higher level.

The electrolyte serves as a medium of ionic conductivity for the transmission of ions between the cathode and anode. Choosing a proper electrolyte is essential for achieving high energy density, power density, and long cycle life. This is also essential for ensuring the excellent safety performance of ZIHCs. Neutral or slightly acidic solutions (such as ZnSO_4_, ZnCl_2_, and Zn[CF_3_SO_3_]_2_) are widely employed as electrolytes in aqueous ZIHCs [42–44]. Unfortunately, the freezing of aqueous electrolytes typically results in a dramatic loss of ionic conduction, seriously restricting the low-temperature application of such ZIHC devices. The voltage window of ZIHCs is restricted to the range of 0–1.8 V using these electrolytes (Table 1). It is accepted that the voltage window could be widened by developing novel electrolytes enabling high-voltage windows. Yang et al. [45] designed a supramolecular gel polymer electrolyte (SGPE) consisting of ZnSO_4_/sodium alginate/poly(vinyl alcohol) for a ZIHC. The SGPE electrolyte demonstrates a wide voltage range of 0.2–2.0 V for ZIHCs. Wu et al. [46] developed a eutectic gel electrolyte containing a ternary deep eutectic solvent (DES) based on Zn(ClO_4_)_2_, acrylamide, and H_2_O. The operating voltage window of the constructed flexible ZIHC was extended to 0–2.2 V resulting from the inhibition of the free water activity in DES. Moreover, the contact of the zinc anode with the liquid electrolyte also causes some issues, such as uncontrolled zinc corrosion, dendrite growth, and undesirable side reactions [47, 48]. The sharp zinc dendrites on the Zn anode may pierce the separator, which will make the cathode and anode electrodes of ZIHCs contact, resulting in a short circuit and shortening the cycle life of the device [49]. An appropriate separator can guide the regular deposition of zinc in the process of charge and discharge, and effectively solve the problem of zinc dendrite formation.

**Table 1:** A summary of current collectors and separators for ZIHCs

Cathode	Electrolyte	Current collector	Separator	Voltage (V)	Capacitance/Current density (F g^−1^/A g^−1^)	Capacity/Current density (mAh g^−1^/A g^−1^)	Energy density (Wh kg^−1^)	Power density (kW kg^−1^)	Refs.
PC800	3 M Zn(ClO_4_)_2_	Stainless steel mesh	Wood pulp/polyester fabric	0–1.9	340.7/0.1	179.8/0.1	104.8	48.8	[38]
RbPC	1 M Zn(CF_3_SO_3_)_2_	Stainless steel mesh	Glass fiber membrane	0.2–1.8	–	247.6/1	178.2	72.3	[50]
NPFCs	1 M Zn(CF_3_SO_3_)_2_	Stainless steel mesh	polypropy-lene	0.2–1.8	207.9/0.1	129.9 /0.1	85.7	15.4	[51]
PSC-A600	1 M Zn(CF_3_SO_3_)_2_	Graphite paper	Whatman filter paper	0.2–1.8	413.3 /0.2	183.7/0.2	147	0.1361	[52]
nPC	2 M ZnSO_4_	Carbon cloth	–	0.15–1.7	–	302 /1	157.6	0.69	[53]
B2S3C	1 M ZnSO_4_·7H_2_O	Stainless-steel net	–	0.2–1.8	–	182.6/0.1	292.2	0.0622	[54]
HPCS-900	3 M ZnSO_4_	Ti foil	Whatman glass fibers	0.1–1.7	–	104.7	90.17	0.0812	[55]
AC-SA	2 M ZnSO_4_	Ti mesh	Glassy fiber membrane	0.15–1.8	436/0.02	200/0.02	134.8	118.4	[56]
PBC-A900	1 M Zn(CF_3_SO_3_)_2_	Stainless steel mesh	Glass fiber	0.2–1.8	321.3/1	–	114.2	0.8	[57]
BGC-750	3 M Zn(CF_3_SO_3_)_2_	Stainless steel foil	Filter paper	0.1–1.8	–	257/0.5	168	61.7	[58]
N, P-OLC	2 M ZnSO_4_	Carbon cloth	Glass fiber paper	0.2–1.8	420.3/0.5	184.5/0.5	149.5	26.7	[59]
N, S-PCD	2 M ZnSO_4_	Carbon paper	Whatman filter paper	0.2–1.8	300.2/0.2	133.4/0.2	106.7	0.16	[60]
NC	PAAm/agar/Zn(CF_3_SO_3_)_2_	Graphite paper	–	0.2–1.8	–	73.4/0.25	61.3	16.5	[61]
PZC-10	2 M ZnSO_4_	Carbon cloth	Glass microfiber filters	0.2–1.8	310/0.5	–	43	137.9	[62]
AC	P(AMPSZn-AAZn)/ZnCl_2_	Carbon cloth	–	0–1.8	458.7 /1	229.4/1	205.3	1.01	[63]
NPC	1 M ZnSO_4_	Carbon paper	–	0–1.8	–	136.2/0.3	81.1	12.8	[64]
AC	Zn(TFSI)_2_/[[Pyr_14_TFSI]_3_]_16_/[AN]_4_	Stainless steel mesh	Glass microfiber membrane	0–2.1	143.3 /0.3	–	84	0.311	[65]
BCF	2 M ZnSO_4_	Carbon paper	Whatman filter separator	0.1–1.8	–	133.5/1	119.7	0.89	[66]
LHPCs	1 M ZnSO_4_	–	–	0.2–1.8	298/0.1	–	135	0.101	[67]
ORC-900	1 M ZnSO_4_	Stainless steel mesh	Whatman glass microfibers	0.2–1.8	308/0.5	–	136.5	0.57	[68]
ZMDPC-700	2 M ZnSO_4_	Stainless steel	–	0.2–1.8	255.6/0.5	–	57.71	4.5	[69]
NPMC2	2 M ZnSO_4_/0.05 M ZnI_2_	Stainless steel foil	Glass fiber	0.2–1.8	–	333.4/0.5	324.8	0.4126	[70]
TWC-2	2 M ZnSO_4_	Carbon cloth	Waterman GF/D glass fiber	0–1.8	363.5/0.5	–	81.8	0.1377	[71]
AC-PHC	1 M ZnSO_4_	Stainless steel	–	0.2–1.8	–	146.4/0.1	117	0.16	[72]
AC-330	1 M ZnSO_4_	Stainless steel	–	0.2–1.8	–	212.5/0.1	170	0.1704	[73]
CDMF	3 M Zn(CF_3_SO_3_)_2_	Stainless-steel mesh	Glassy fibrous	0–1.8	–	180/0.2	106.7	13.4	[74]
C-Xs	3 M ZnSO_4_	Stainless steel	paper fiber membrane	0.2–1.8	–	121.7/0.05	109.3	15.6	[75]
NPCNs	1 M Zn(CF_3_SO_3_)_2_	–	Paper fiber membrane	0.2–1.8	–	204.7/0.1	143	0.0716	[76]
RHC-850	3 M Zn(CF_3_SO_3_)_2_	Foamed nickel	Glass fiber paper	0.1–1.8	149.8/0.2	–	58.6	0.1678	[77]
PCCs-3	1 M Zn(CF_3_SO_3_)_2_	–	Whatman glass microfibers	0.2–1.8	345/0.5	–	119.02	0.35517	[78]
3DPC	2 M ZnSO_4_	Carbon cloth	Non-woven fabric	0.2–1.8	–	194/0.5	156	0.004	[79]
NSC-6	Bacterial cellulose /ZnSO_4_	Graphite paper	–	0.2–1.8	–	113/0.5	29.9	5.673	[80]
BCN-Mg	2 M ZnSO_4_	Stainless net	Glass fiber	0.2–1.8	–	130.7/0.1	104.5	0.08	[81]
CMF-800	1 M ZnSO_4_	Stainless steel mesh	Glass fiber (GF/F, Whatman)	0.2–1.8	249.7/0.5	–	54	14.65	[82]
NHPC	0.5 M Zn(CF_3_SO_3_)_2_	Graphite paper	–	0.2–1.8	183.8/0.1	–	71.8	10.3	[83]

RbPC, porous carbon derived from tetra-alkali metal pyromellitic acid salts (PMA4M, M = Rb); NPFCs, N, P dual doped foamy-like carbons; CSAC, coconut shell activated carbon; PSC-A600, pencil shaving derived porous carbon activated by KOH at 600 °C; nPC, pyridinic nitrogen enriched porous carbon; B_2_S_3_C, B, S co-doped spongy-like HPC; AC-SA, activated carbon with sodium alginate binder; PBC-A900, porous bamboo carbon activated by KOH at 900 °C; BGC-750, bone glue-derived carbon second carbonized at 750 °C; N, P-OLC, N, P co-doped onion-like carbon; N, S-PCD, N, S co-doped porous carbon dodecahedra; PZC-10, ZIF-8@ phosphatidylcholine (ZIF-8/PC: 10/1) derived carbon; BCF, B, N and O co-doped flower-like carbon; LHPCs, lignin-derived hierarchical porous carbons; ZMDPC, co-doped zinc-based MOF derived porous carbon; NPMC, N/P co-doped monolithic hierarchical porous carbon; TiN/CNTs@CC and TWC, carbon cloth-supported TiN-coated carbon nanotube composites; AC-PHC, pitch coke derived activated carbons; C_DMF_, O/N-decorated porous carbon; C-Xs, porous carbon nanosheets prepared by dual-template strategy combined K_2_CO_3_ activation from anthracene molecules, RHC-850, rice husks derived carbon at a carbonization temperatures of 850 °C; PCCs, porous carbon cages; BCN-Mg, B, N co-doped carbon nanosheets; CMFs, carbon micro-foams

**Fig. 2:**
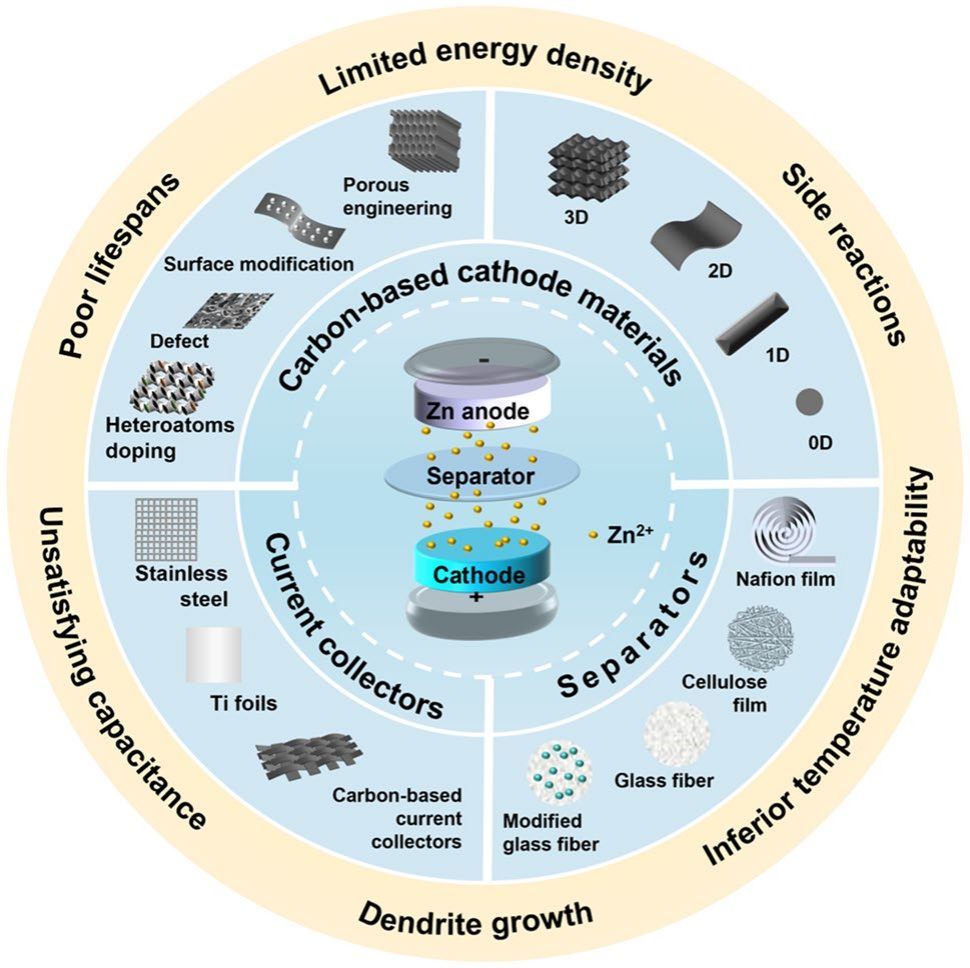
The performance improvement approaches of carbon-based materials with different dimensions, the application of current collectors and separators in ZIHCs, and the challenges faced by ZIHCs today

In this review, we briefly summarize the recent advances in the research on advanced carbon cathode materials with multi-dimensional structures for ZIHCs, focusing on their structural design and electrochemical features. Current collectors and separators used in ZIHCs are showcased for the first time in a review paper (Fig. 2). Lastly, we propose the challenges and prospects of ZIHCs, hoping to promote the innovation of optimizing carbon-based cathode materials and developing novel ZIHCs.

## Charge Storage Mechanisms and Carbon Materials with Multi-dimensional Structures

**Fig. 3:**
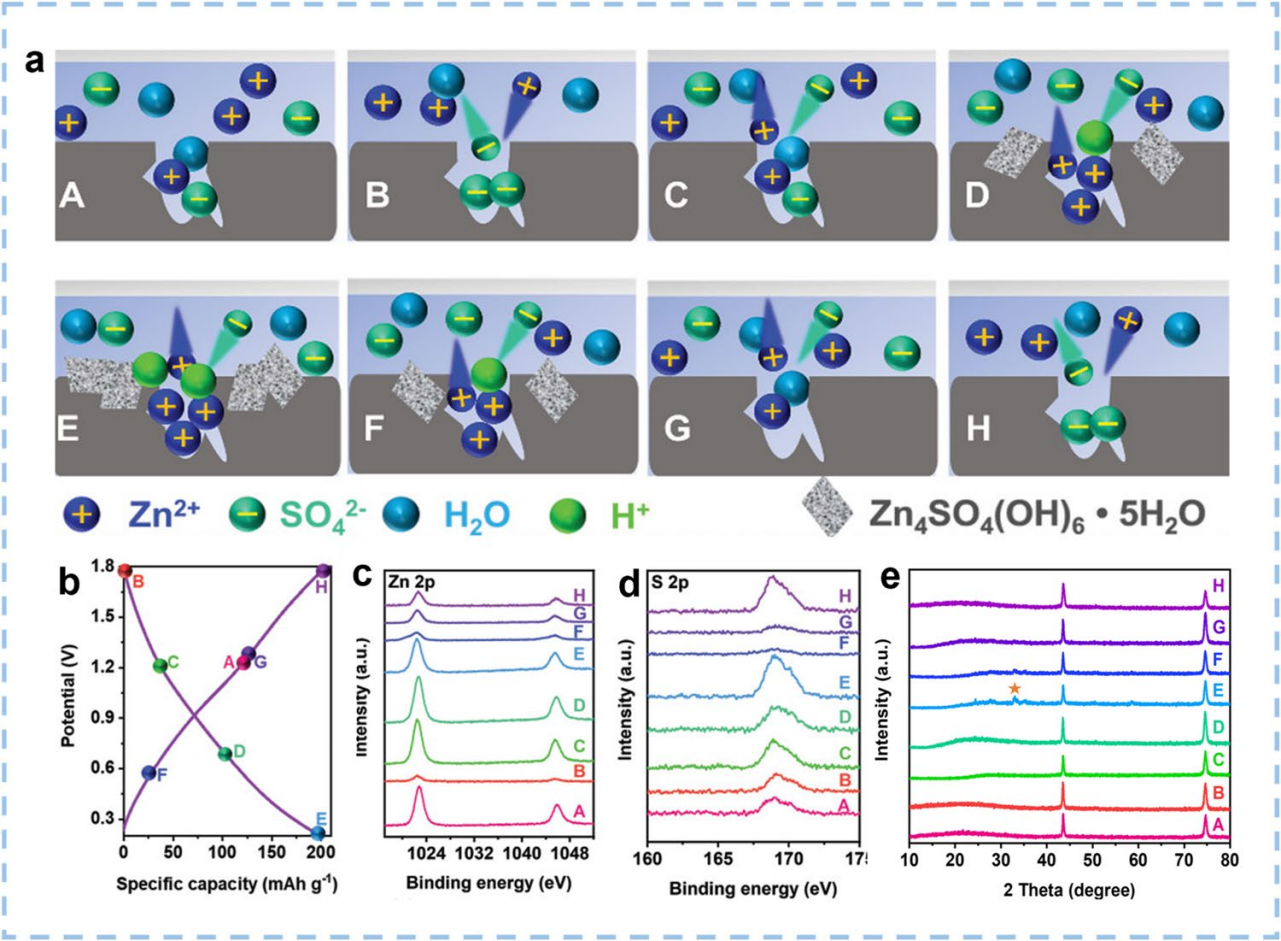
The charge storage mechanism of ANHPC-2 in ZnSO_4_ aqueous electrolytes at different charge/discharge states. **a** Schematic illustration of the charge storage mechanism for ANHPC-2 in 2 mol L^−1^ ZnSO_4_ electrolyte. **b** Typical GCD curve at 0.5 A g^−1^ and corresponding ex situ. **c** Zn 2*p*, **d** S 2*p*, XPS spectra of ANHPC-2 cathode at the selected voltage points. **e** Ex situ XRD patterns of ANHPC-2 cathode at different stages. Reproduced with permission [84]. Copyright 2022, Wiley-VCH

Among various cathode materials, carbon materials are the most widely studied and applied in ZIHCs. Combining electrochemical theoretical analysis and the phase change of the reaction products on the electrode surface, discussion on the charge storage mechanism of the carbon cathode is blooming. Wang et al. [84] prepared the nitrogen-doped hierarchical porous carbon materials (ANHPC-x, where x represents the mass ratio of KOH to nitrogen-doped hierarchical porous carbon. Eight points in the typical first discharge and second charge curves were selected to thoroughly investigate the charge storage mechanism of ANHPC-2 in ZnSO_4_ electrolyte (Fig. 3a). Typically, carbons store charge through the synergistic effect of anion and cation co-adsorption mechanisms at different potentials and reversible adsorption/desorption. The reversible dual-ion adsorption of cation and anion occurred in different potential ranges (Fig. 3a). Cation (Zn^2+^, H^+^) adsorption/desorption is the dominant process at low potentials, accompanied by the formation/dissolution of Zn_4_SO_4_(OH)_6_·5H_2_O (Eq. 2) [32, 85]. The storage capacity for Zn^2+^ ions of carbon materials can be raised by the formation of Zn_4_SO_4_(OH)_6_·5H_2_O.

2 $$ {\text{4Zn}}^{{2 + }} {\text{ + 6OH}}^{ - } {\text{ + SO}}_{{4}}^{{{2} - }} {\text{ + 5H}}_{{2}} {\text{O }} \leftrightarrow {\text{ Zn}}_{{4}} {\text{SO}}_{{4}} {\text{(OH)}}_{{6}} \cdot {\text{5H}}_{{2}} {\text{O}} \downarrow $$

As shown in Fig. 3b, c, from state B to E and state A to B, the intensity of Zn 2*p* first increased in the first discharge process and then decreased during the second charge process, indicating that Zn^2+^ adsorbed onto ANHPC-2 and Zn^2+^ desorption from the surface of ANHPC-2. Notably, the intensity of Zn 2*p* at state E is higher than other potential states, revealing the Zn^2+^ was widely adsorbed in the carbon cathode during the deep discharge (Fig. 3c). While at high potentials, anion (SO_4_^2−^) adsorption/desorption is the dominant process. From state E to G, the S 2*p* signal presents decreasing tendency, implying the content of SO_4_^2−^ is gradually decreased (Fig. 3b, d). Moreover, the intensity of S 2*p* at state H is higher than state G, demonstrating SO_4_^2−^ was further adsorbed on the ANHPC-2 at high potential region (Fig. 3d). The intensity of S 2*p* displays the highest content at state E, attributing to the highest content of Zn_4_SO_4_(OH)_6_·5H_2_O at state E. The characteristic peak (32.5°) of the Zn_4_SO_4_(OH)_6_·5H_2_O byproduct was detected at states E (Fig. 3e), which is attributed to the pH change of electrolyte at the low potential region. The electrochemical adsorption of H^+^ creates a dynamic local alkaline environment near the electrode surface, where OH^−^ anions are strongly coordinated with Zn^2+^, leading to the formation of Zn_4_SO_4_(OH)_6_·5H_2_O [84, 86].

Wang et al. [50] described the charge storage mechanism of porous carbon materials in Zn(CF_3_SO_3_)_2_ aqueous electrolyte through a series of ex-situ characterization techniques (ex-situ X-ray diffraction (XRD), Raman spectra, X-Ray photoelectron spectroscopy (XPS), and scanning electron microscope (SEM)) and in-situ electrochemical quartz crystal microbalance (EQCM). Similar to the mechanism in aqueous ZnSO_4_ electrolytes, the adsorption/desorption of Zn^2+^ ions mainly occur in the low voltage range. The adsorption/desorption of CF_3_SO_3_^−^ primarily occur in the high voltage range, and the adsorption/desorption of CF_3_SO_3_^−^ and Zn^2+^ occur simultaneously in the medium voltage region. The reversible deposition and dissolution of Zn(CF_3_SO_3_)_2_[Zn(OH)_2_]_3_·5H_2_O that occurred during the charge/discharge processes is beneficial to the adsorption of Zn^2+^. Their subsequent research reported a supplementary insight: the adsorption of Zn^2+^ raises the defect content of the material, particularly after the material has been discharged sufficiently [84]. The adsorption of Zn^2+^ and H^+^ is proven using electrochemical techniques. The co-adsorption of H^+^ and Zn^2+^ enhances the charge storage capability of oxygen-rich porous carbon cathodes since oxygen has a positive effect on enhancing the adsorption ability of zinc ions and protons [38].

**Fig. 4:**
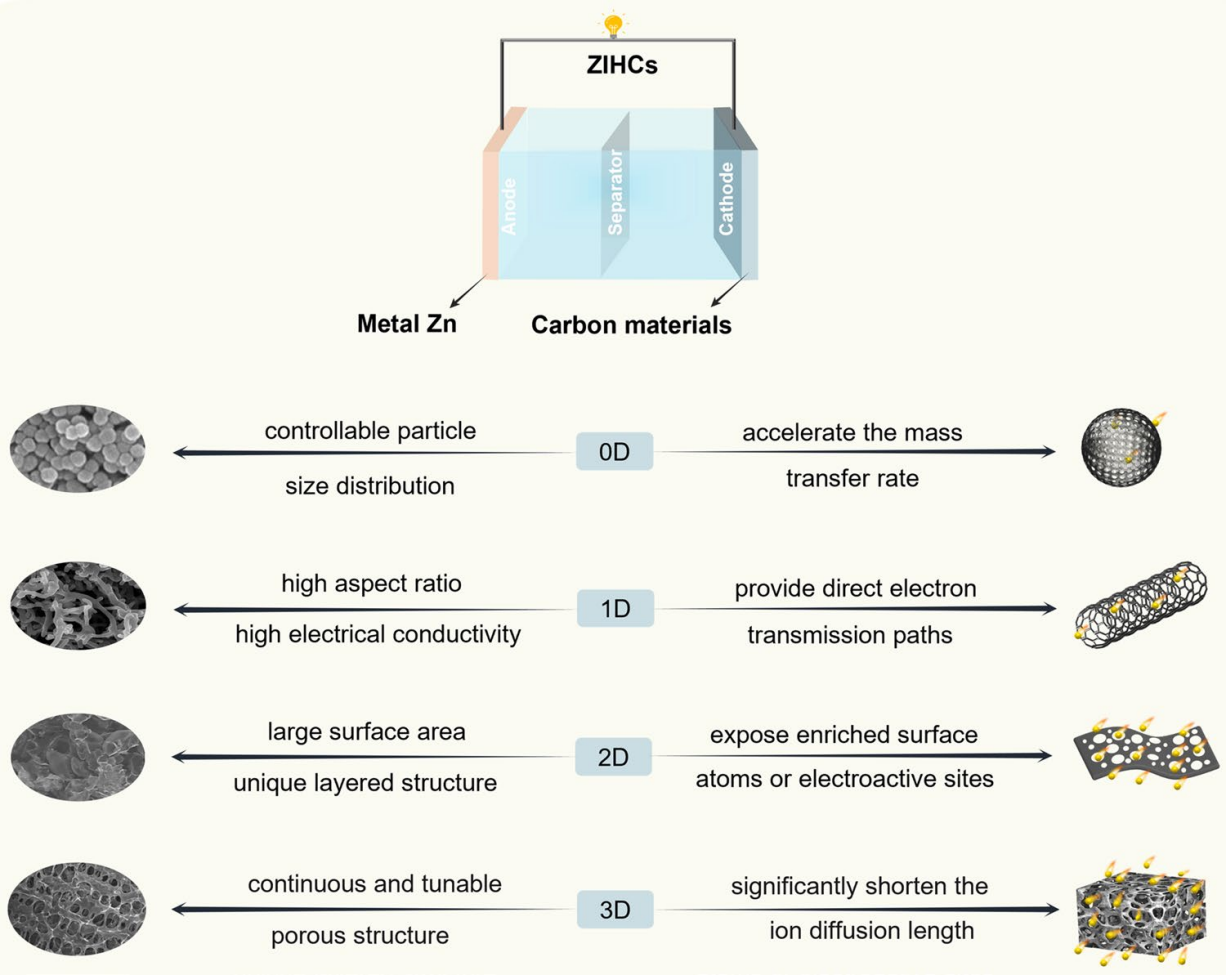
Overview of carbon-based cathode materials with different dimensions for ZIHCs

It has always been an intriguing research point to improve the electrochemical performance of carbon materials through structural optimization. If the pore surface is efficiently utilized to build an electric double layer, the capacitive performance of carbon materials mainly depends on the EDL capacitance, according to the formula (Eq. 3),

3 $$ C = \frac{{\varepsilon_{r} \varepsilon_{0} A}}{d} $$

where *C* is the EDL capacitance, *ε*_*r*_ is the electrolyte dielectric constant, *ε*_*0*_ is the dielectric constant of the vacuum, *d* is the effective thickness of the double layer, *A* is the electrode surface area. Theoretically, a high SSA usually results in a high specific capacitance. However, current research shows that the specific capacitance of carbon materials is not strictly positively correlated with the SSA, because not all pores in the material can store charge. Although some pores contribute to the SSA, they are difficult for the electrolyte to enter due to factors such as twisted pore channels or small pore size [87]. It can be seen that the pore structure of carbon materials is a vital parameter, where proper pore size and distribution allow for higher storage efficiency for zinc ions. [Zn(H_2_O)_6_]^2+^, as the main solvated structure in low-concentration Zn electrolytes, has been identified as the main charge carrier for ZIHCs during charge/discharge processes [88]. When the pore sizes of nano porous carbons are larger than 6.8 Å, [Zn(H_2_O)_6_]^2+^ with an ion size of 5.5 Å is easy to diffuse, and hence greatly ameliorates the specific capacitance and rate capability of ZIHCs [89, 90]. Therefore, when designing strategies to optimize the electrochemical performance of carbon materials, in addition to the high specific surface area, the pores and pore distribution of the materials should also be considered. Carbon materials are divided into zero-dimensional (0D) carbon materials such as carbon spheres, one-dimensional (1D) carbon materials, such as carbon nanotubes and carbon fibers, two-dimensional (2D) carbon materials, such as graphene and carbon nanosheets, and three-dimensional (3D) carbon materials, such as 3D porous carbon, 3D graphene, and hierarchically porous carbon (Fig. 4). In this section, the structures, electrochemical properties, and recent progress of carbon materials with different dimensions for ZIHCs are discussed, with a particular emphasis on methods to improve the capacitance of carbon materials.

### 0D Carbon Materials

0D carbon nanomaterials refer to substances that are at the nanoscale in all three dimensions, and the size is between 1 and 100 nm [91]. The morphology of 0D carbon nanomaterials here refers to carbon nanoparticles. They have the characteristics of accelerating the mass transmission rates because of their small nano size, unique spherical structures, and the controllable particle size distribution. Carbon spheres are the most explored 0D carbon materials for *ZIHCs*.

#### Carbon Spheres

Carbon spheres (CSs) with uniform particle sizes possess some distinct advantages, such as good conductivity, adjustable porosity, and controllable particle size distribution [92–95]. These CSs therefore exhibit great utilitarian value for being employed as electrode materials for ZIHCs. According to their structural characteristics, CSs can be divided into hollow CSs (HCSs) [96, 97], yolk-shell structured CSs [98, 99], core–shell structured CSs [100, 101] etc. HCSs are widely used as cathodes for ZIHCs at this stage. The hollow structure can accelerate the mass transfer, which is conducive to the diffusion of electrolyte ions, and relieve the volume expansion during the repeated charge and discharge processes.

Various synthetic strategies have been developed for preparing HCSs, the SiO_2_ hard template method is a conventional synthesis method. Specifically, a layer of carbon precursor is coated on the silica spheres, then the precursor is converted into carbon after annealing, and finally the SiO_2_ is removed by hydrofluoric acid (HF) or sodium hydroxide (NaOH) etching [102]. Resorcinol–formaldehyde (RF) is the representative carbon precursor in the recently reported literature. Chen et al. [103] synthesized hollow mesoporous carbon spheres (HMCSs) from the core–shell SiO_2_@RF nanospheres. The obtained HMCSs show an average particle diameter of 350 nm and an SSA of 800 m^2^ g^−1^. To explore the electrochemical performance of the HMCSs, a ZIHC, in which the HMCSs served as the both cathode and protective layer of the zinc anode was assembled. The device exhibits a capacitance of 212.5 F g^−1^ at 0.2 A g^−1^ and a 99.4% capacitance retention rate after 2500 cycles at 2 A g^−1^. Similarly, Fei et al. [104] designed hollow bowl-like carbon (HBC) spheres with abundant mesopores by an in situ hard-template route. The silica spheres and RF were used as the template and carbon precursor, respectively. The HBC spheres exhibit a diameter of 300 nm and an SSA of 791.5 m^2^ g^−1^. Besides, tetraethyl orthosilicate (TEOS), one of the raw materials, is the key to preparing mesoporous SiO_2_ templates. By changing the mass ratio of (R + F)/TEOS, the morphology of the obtained carbon can be adjusted to a bowl or sphere. (R + F)/TEOS = 2 or 0.5 corresponds to whether the shell is thick or thin when the ultimate carbon morphology is a hollow sphere. Experimental results show that the HBC spheres are superior to HCSs in packing density, electronic conductivity as well as volumetric capacity. Benefiting from their unique hollow bowl-like structures, HBC spheres show a specific capacity of 95.4 mAh g^−1^ at a current density of 0.1 A g^−1^ when employed as the cathode materials of ZIHCs.

**Fig. 5:**
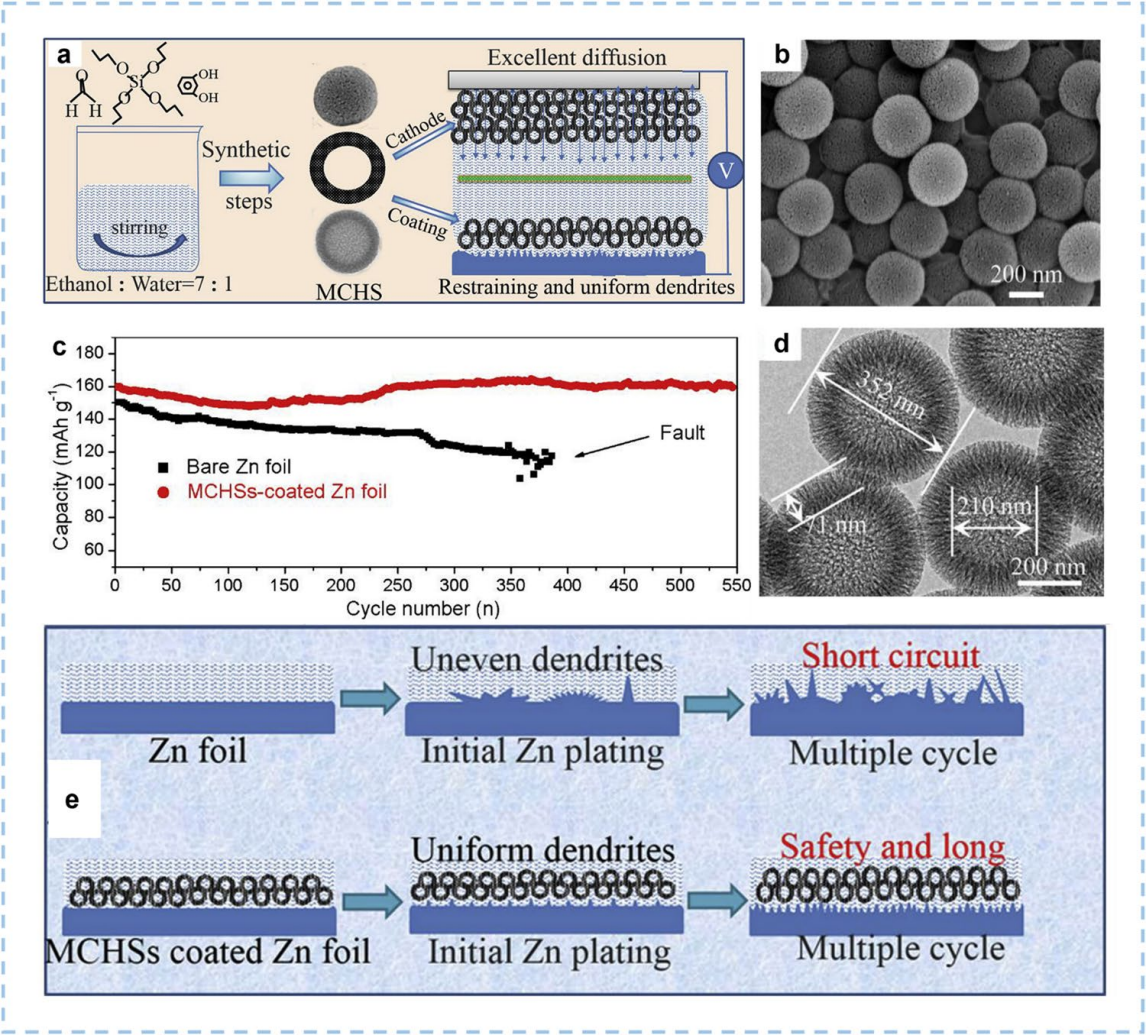
The fabrication process and structure of MCHSs and comparison between bare Zn foil and Zn foil coated with MCHSs. **a** Schematic illustration for the synthesis of MCHSs. **b**, **d** SEM images of MCHSs. **c** Cycling performance of the MCHSs-coated Zn∥MCHSs and Zn∥MCHSs at 0.1 A g^−1^. **e** Schematic illustrations of morphology evolution for bare and MCHSs-coated Zn foils during Zn stripping/plating cycling. Reproduced with permission [105]. Copyright 2020, Elsevier

HCSs can also be used as a coating layer to adjust the distribution of dendrite/protrusion nucleation sites on the surface of Zn anodes. This opens a universal approach to regulating zinc deposition. Liu et al. [105] synthesized mesoporous carbon hollow spheres (MCHSs) which can be used as the cathode material and the coating layer of Zn foil simultaneously (Fig. 5a). The MCHSs display an average diameter of 352 nm and an SSA of 1275 m^2^ g^−1^ (Fig. 5b, d). Compared with bare Zn, the MCHSs coating can guide the uniform distribution of zinc dendrites, which is attributed to the well-proportioned charge distribution and rapid electron transportation pathways (Fig. 5e). Thus, the cycling stabilities of MCHSs-coated Zn//MCHSs ZIHCs are better than those of Zn//MCHSs ZIHCs, which exhibits a 96% capacitance retention rate after 10,000 cycles at 1 A g^−1^ (Fig. 5c). Furthermore, the capacity of MCHSs is 174.7 mAh g^−1^ at 0.1 A g^−1^, and it can maintain at 96.9 mAh g^−1^ when the current density increases by 100 times.

In general, CSs with hollow structures have shown great promise in the application of ZIHCs electrode materials. The research that deserves more attention is the optimization of their synthetic route. The synthesis methods that have been reported often involve pre-synthetic templates, complex multi-step procedures, or strict reaction conditions. This is undoubtedly time-consuming and poses a threat to the practical application of CSs. However, the CSs with uniform pore size distribution and controlled properties are beneficial for the production of ZIHCs with product quality.

### 1D Carbon Materials

Carbon materials with 1D nanostructures, long linear chains of carbon atoms known as "linear carbons" or carbynes, have become a candidate for ZIHC electrode materials [106]. The high aspect ratio, high electronic conductivity, and excellent mechanical deformability of 1D carbon materials and thus can provide direct electron transportation pathways when employing as cathode materials of ZIHCs. Furthermore, their excellent mechanical properties make them more attractive for flexible or wearable electronics compared with traditional planar structures [107–109]. Carbon nanotubes and carbon nanofibers are representative 1D carbon materials.

#### Carbon Nanotubes

Carbon nanotubes (CNTs) are considered a derivative of both carbon fibers and fullerene [110]. CNTs have emerged as promising electrode materials owing to their excellent properties, such as high tensile strength, ultra-lightweight, unique electronic structures, high chemical and thermal stability, and large surface area (up to 1300 m^2^ g^−1^) [40]. Sun et al. [111] constructed a zinc-ion micro-supercapacitor (ZmSC) by integrating a capacitor-type CNT micro-cathode and a battery-type zinc micro-anode. The prepared ZmSC exhibits an area capacitance of 83.2 mF cm^−2^ at 1 mA cm^−2^, an energy density of 29.6 μWh cm^−2^ and a power density of 8 mW cm^−2^. Furthermore, the conductivity of active materials can be boosted by using CNTs as conductive substrates. Zhang et al. [112] demonstrated a quasi-solid-state Zn-ion hybrid fiber-shaped capacitor (ZnFC) in which the conductive reduced graphene oxide/carbon nanotubes composite fibers served as capacitor-type positive electrodes. The ZnFC exhibits a specific capacitance of 104.5 F cm^−3^ at a current density of 400 mA cm^−3^ and an energy density of 48.5 mWh cm^−3^ at a power density of 179.9 mW cm^−3^. Similarly, Lin et al. [113] synthesized a core–shell-structured multi-walled carbon nanotube@graphene oxide nanoribbon (NP-MWCNT@GONR) by unzipping and activating processes. The NP-MWCNT@GONR was prepared by ZnCl_2_ activation, while its control sample, MWCNT@GONR, was prepared without activation. The SSA of the NP-MWCNT@GONR is 54.2 m^2^ g^−1^, which is slightly higher than the MWCNT@GONR (49.0 m^2^ g^−1^). When used as the cathode material of ZIHCs, the NP-MWCNT@GONR exhibits a high capacitance of 185.3 F g^−1^ at a current density of 0.1 A g^−1^, while the corresponding capacitance of MWCNT@GONR is 152.9 F g^−1^. The energy density of the as-assembled NP-MWCNT@GONR//Zn ZIHC in 3 M Zn (CF_3_SO_3_)_2_ liquid electrolyte is 90.09 Wh kg^−1^ at 95 W kg^−1^, and the power density is 19 kW kg^−1^ at an energy density of 30.55 Wh kg^−1^.

**Fig. 6:**
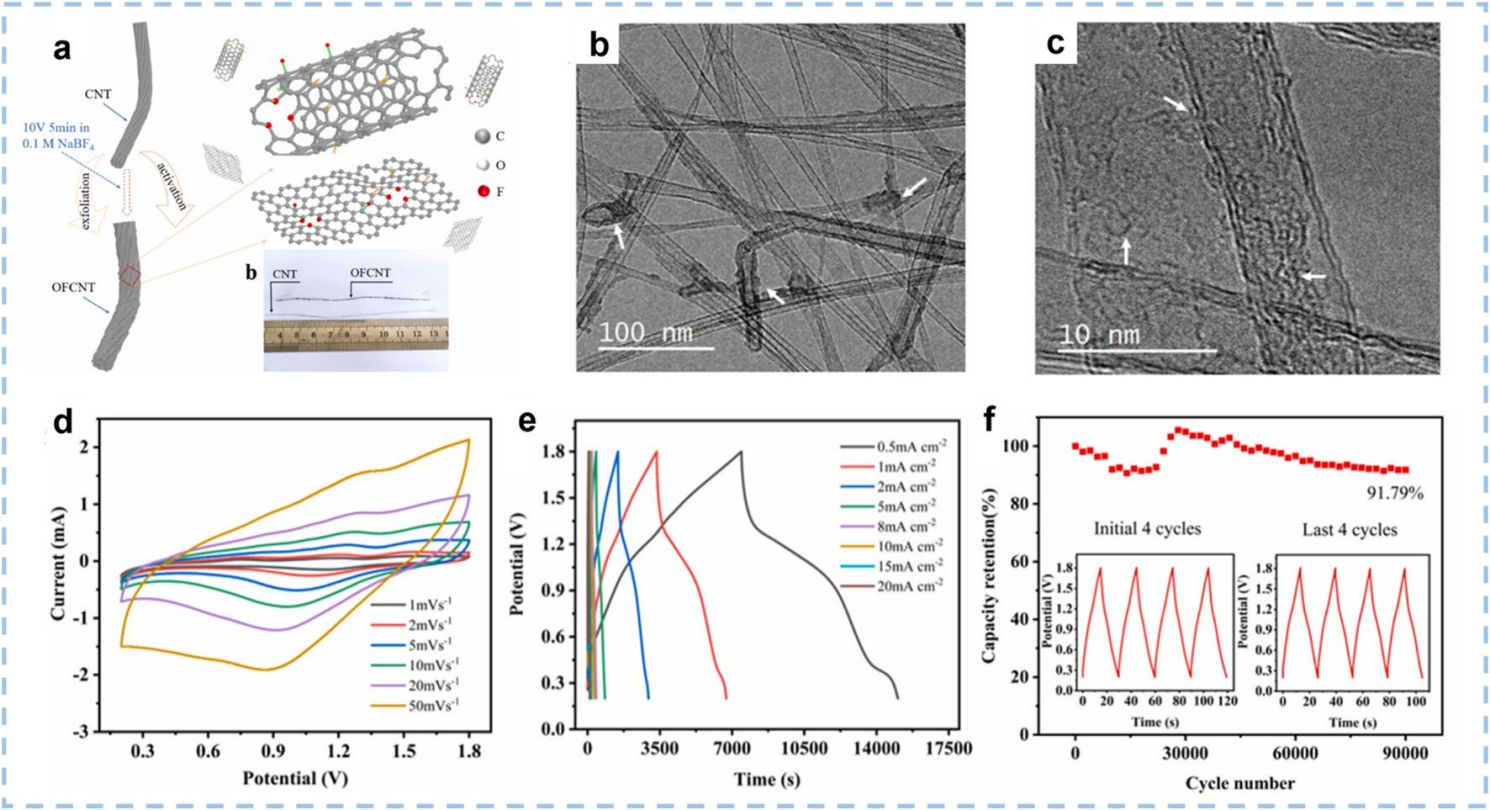
The synthesis route, morphology, and structure of the OFCNT-5 sample and the electrochemical performance of Zn//OFCNT-5 device. **a** Diagram of the synthesis route of OFCNT sample. **b** TEM image and c the relevant HRTEM image of the OFCNT-5 fiber bundles sample. **d** CV curves of Zn//OFCNT-5 device at various scan rates of 1–50 mV s^−1^. **e** GCD curves of Zn//OFCNT-5 device under various areal current densities of 0.5–20 mA cm^−2^. **f** Long-cycling stability of Zn//OFCNT-5 device over 90,000 cycles under the current density of 1 mA cm^−2^, the inset images depict the initial 4 cycles and the last 4 cycles of GCD curves. Reproduced with permission [117]. Copyright 2022, Elsevier

Low capacitance is the major drawback of CNTs despite their high conductivity and excellent cycling stability. Two effective strategies can be used to overcome this constraint. One possible solution is to induce heteroatoms, which enable superior aqueous Zn^2+^ ions storage capability owing to the introduction of pseudocapacitive effects. The role of sulfur (S), nitrogen (N), and phosphorus (P) elements doping in CNTs has been extensively studied [51, 114, 115]. Li et al. [116] synthesized sulfur-incorporated, nitrogen-rich porous carbon nanotubes (SN-PCNTs) with a hollow structure. The SN-PCNTs exhibit an SSA of 589.2 m^2^ g^−1^ and a typical mesoporous feature with pore size ranging from 3 to 5 nm. The control sample of N-PCNTs shows a similar mesoporous structure and a relatively smaller SSA of 381.5 m^2^ g^−1^. This indicates that the incorporation of S could increase the SSA of carbon materials. Moreover, the density functional theory calculation (DFT) results indicate that the S incorporation can significantly enhance the adsorption of Zn^2+^ ions and modulate the electron transfer behavior of the CNTs. Consequently, aqueous ZIHC based on the SN-PCNTs cathode delivers a high energy density of 95.9 Wh kg^−1^ at 125 W kg^−1^ and a superb power density of 19,170 W kg^−1^ at 21.3 Wh kg^−1^, as well as an ultralong lifespan of up to 25,000 cycles with a high capacity retention of 93.5%. There have been few reports on the introduction of fluorine (F) dopant, the element with the highest electronegativity, into carbon lattices to date. Chen et al. [117] prepared an OFCNT fiber electrode with abundant oxygen/fluorine functional groups through a simple electrochemical exfoliation and activation method (Fig. 6a–c). The as-fabricated ZIHC based on an OFCNT-5 cathode achieves an ultrahigh areal capacitance of 1556.8 mF cm^−2^ (corresponding volumetric capacitance of 593.1 F cm^−3^), impressive long-cycling stability with a capacity retention rate of 91.8% after 90,000 cycles (Fig. 6d–f). Besides, such a device also delivers a high areal energy density of 553.53 μWh cm^−2^ (210.86 mWh cm^−3^) and a great power density of 26.83 mW cm^−2^ (10.22 W cm^−3^). The very favorable performance of OFCNT-5 could be attributed to the synergistic effect of oxygen/fluorine functional groups, which led to abundant C–OH bonds and semi-ionic C–F bonds attaching to the OFCNT electrode, thereby facilitating the strong interaction with Zn^2+^ ions to contribute pseudocapacitance. It is approved that the oxygen termination could enhance the adsorption of Zn^2+^ and H^+^ ions.

Combining CNTs with a polymer or metal oxide is another well-acknowledged approach for improving the specific capacitance and rate performance of devices. For example, Li et al. [118] synthesized hydroxylated carbon nanotube (h-CNTs)/polyaniline (PANI) nanocomposites cathode by in-situ polymerization of PANI on h-CNTs. Incorporating the capacitive energy storage mechanism of h-CNTs and the redox reaction energy storage mechanism of PANI, the nanocomposites exhibited superior comprehensive electrochemical performances. The h-CNTs possess the highest capacity of 153 mAh g^−1^ due to their superior hydrophilicity and chemical adsorption ability of Zn^2+^ ions. The as-assembled ZIHC based on h-CNTs/PANI nanocomposite cathode delivers cyclability with 90% capacitance retention even after 600 cycles of repeated bending. Manganese-based materials have higher theoretical capacitance (e.g., the theoretical specific capacitance of MnO_2_ is 1370 F g^−1^) and better operation voltage than CNTs, but their electrical conductivity is intrinsically poor [119]. The construction of manganese-based materials/CNTs composites can combine their respective advantages, which will significantly improve the electrochemical performance of CNT-based ZIHCs. The specific capacitance of the composite electrode is directly proportional to the loading content of MnO_2_. Wang et al. [120] constructed a ZIHC based on manganese dioxide-carbon nanotubes (MnO_2_-CNTs) as a battery‐type cathode and MXene (Ti_3_C_2_T_x_) as a capacitor‐type anode in an aqueous electrolyte. The as-assembled ZIHC exhibits a specific capacitance of 115.1 F g^−1^, an energy density of 98.6 Wh kg^−1^ at a power density of 77.5 W kg^−1^, and a power density of 2480.6 W kg^−1^ at an energy density of 29.7 Wh kg^−1^. The experimental results show that when the weight ratio of CNTs to MnO_2_ is up to 4/3, the MnO_2_-CNTs electrode exhibits the largest specific capacity at the same current density.

#### Carbon Fibers

Recent years, extensive research has been conducted on carbon fibers (CFs) as one of the freestanding electrode materials for ZIHCs. Their excellent characteristics, such as low cost, high electrical conductivity, and exceptional flexibility, as well as their superior thermal stability and light weight, accelerate the charge storage capability [121, 122]. Surface modification of CFs can improve their electronic conductivity, hydrophilicity, and enhance the chemical adsorption of metal ions. He et al. [123] designed flexible oxygen-enriched carbon fiber films via a facile route. ZIHCs with such carbon fiber cathodes achieve excellent energy and power densities of 97.7 Wh kg^−1^/9.9 kW kg^−1^. Oxygen-containing functional groups on carbon fibers not only significantly enhance their wettability toward aqueous electrolytes, but also greatly facilitate the chemical adsorption of Zn^2+^ ions, resulting in enhanced capacities. Li et al. [86] designed fibrous carbon cathodes with a hierarchical porous structure and O/N heteroatom functional groups. The as-assembled ZIHCs show a high gravimetric capacity of 156 mAh g^−1^, a high energy density of 127 Wh kg^−1^ and a high power density of 15.3 kW kg^−1^. Mechanism investigation reveals that the charge–discharge processes of fibrous carbon cathodes involve cation adsorption/desorption and Zn_4_SO_4_(OH)_6_·5H_2_O formation/dissolution at low voltage and anion adsorption/desorption at high voltage.

**Fig. 7:**
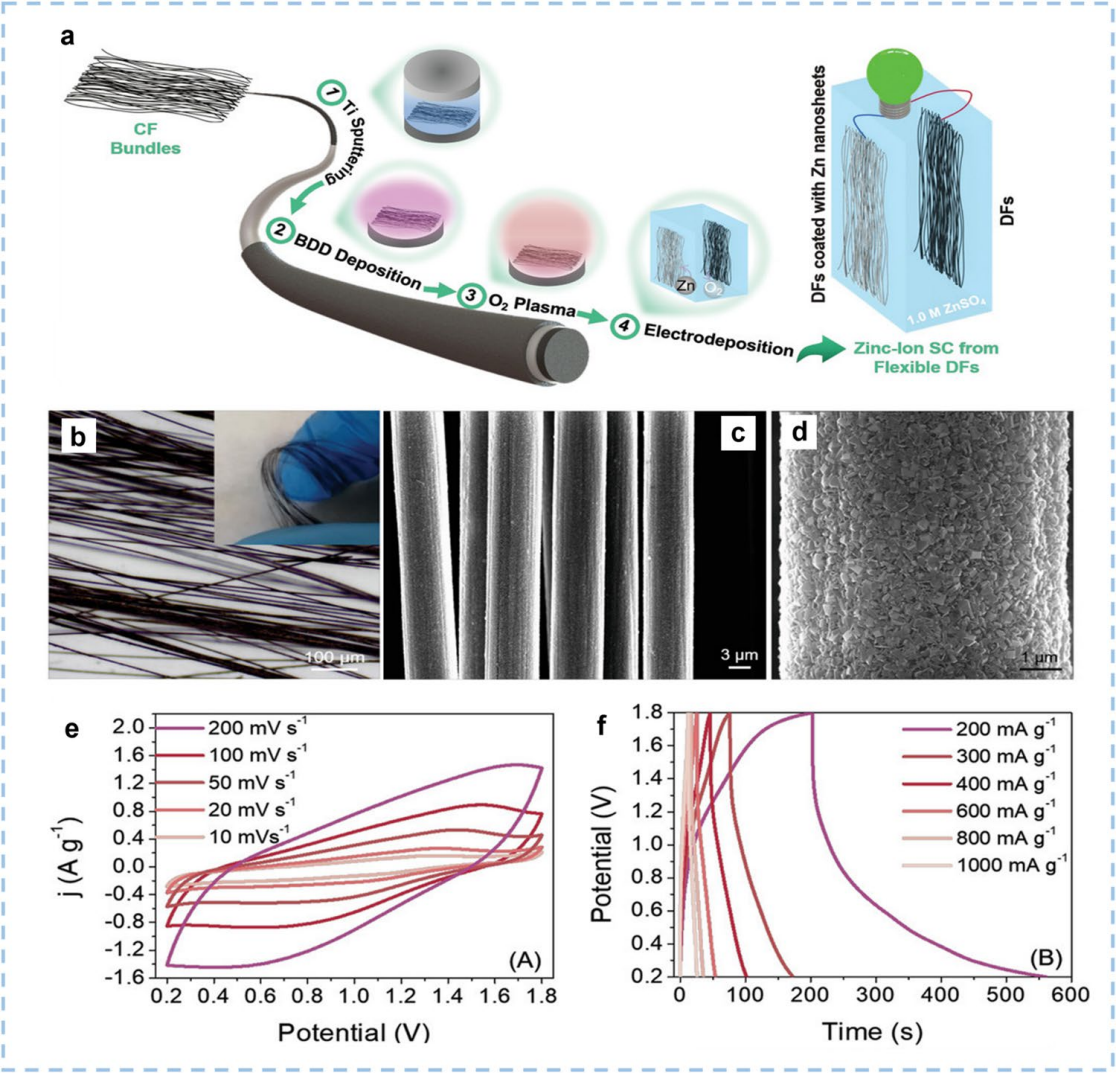
The synthesis procedure, morphology, and structure of the flexible DFs and electrochemical performance of the as-assembled zinc-ion SC in 1 M ZnSO_4_. **a** Schematic illustration of DFs. **b** Optical micrograph with an inset digital photograph. **c**, **d** FESEM images (secondary electron mode). **e** CV curves at different scan rates, **f** GCD curves at different current densities. Reproduced with permission. Reproduced with permission [121]. Copyright 2020, Wiley-VCH

Typically, active materials should be coated on a current collector, such as stainless-steel mesh or Ti foil. Such a manufacturing process is not only complicated but also inevitably introduces electrochemical inert binders into the system. Therefore, it is preferable to construct abundant active adsorption sites on the surface of the CF-shaped current collector and then directly use it as a flexible cathode for ZIHCs. He et al. [124] introduced the high pyridine/pyrrole nitrogen-doped and carbonyl-functionalized nanosheets on flexible electrospun porous carbon nanofibers film. The final product is labeled as N-OPCNF. This composite material avoids the use of current collectors, and electrodes with different mass loadings could be obtained by adjusting the thickness of the N-OPCNF film. After exfoliating the high pyridine/pyrrole nitrogen-doped and carbonyl functionalized nanosheets, the structure of the carbon nanofibers was partially opened, allowing Zn^2+^ ions to accumulate and diffuse into the whole carbon nanofibers. The specific capacity of the N-OPCNF electrode obtained by this strategy is increased to 136 mAh g^−1^. ZIHC based on N-OPCNF cathode shows a high energy of 98.28 Wh kg^−1^, a high power density of 33.2 kW kg^−1^, and ultralong-term cycle stability that remains at 99.2% of initial capacity at 40 A g^−1^ after 200,000 cycles. Jian et al. [121] designed and synthesized flexible diamond fibers (DFs, a fibrous core/shell structure of diamond/carbon fibers) by overgrowing carbon fibers with a thin boron-doped diamond layer by chemical vapor deposition (Fig. 7a–d). The DF has a high surface area of 3068.48 cm^2^ g^−1^. The composites simultaneously possess the high electronic conductivity and flexibility of CFs, and the inherent properties of B-doped diamond fibers (such as wide potential window, long-term stability, environmental friendliness, and high electrochemical activity). Using these diamond fibers as the cathode, the as-constructed ZIHCs display a specific capacitance of 246.1 F g^−1^ at the current density of 200 mA g^−1^ and an energy density of 70.7 Wh kg^−1^ at a power density of 709.0 W kg^−1^ (16.2 Wh kg^−1^ at a power density of 4395.3 W kg^−1^). These values are significantly higher than those reported for DFs and CFs-based SCs so far (Fig. 7e–f).

More importantly, CFs can also be used as ideal substrates for pseudocapacitive electrode materials. Anhydrous vanadium pentoxide (V_2_O_5_) has drawn considerable attention [125–127], especially in the electrochemical field, by virtue of its highest theoretical Zn-storage capacity of 589 mAh g^−1^ (based on the two-electron redox center (vanadium)). However, the intrinsic drawbacks of V_2_O_5_ present in the poor cycling stability and capacity decay at low current densities [128, 129]. The combination of CFs and V_2_O_5_ is a potential solution [130, 131]. Xu et al. [122] designed a new vanadium-based V_2_O_5_ combined with carbon fiber cloth (V_2_O_5_-CFC) cathode by electrospinning and high-temperature calcination. The CFC can enhance the electrical conductivity of V_2_O_5_. Besides, V_2_O_5_-CFC can avoid the aggregation of V_2_O_5_ nanosheets and buffer the volume expansion in the charge/discharge process. As a result, the V_2_O_5_-CFC cathode shows a higher charge/discharge capacity of 185.8 mAh g^−1^ at 1 A g^−1^ than the control sample of pure V_2_O_5_ (133 mAh g^−1^ at 1 A g^−1^). Using this binder-free and current collector-free cathode, the as-fabricated zinc-ion battery shows outstanding reversible capacities for zinc ion storage and excellent flexibility.

In summary, 1D carbonaceous materials like CNTs and CFs have tubular or fibrous structural characteristics. Not only do they have high electrical conductivity, and high chemical stability, but they can also provide a direct electron transmission path. Compared with 2D and 3D carbon, the bottleneck of 1D carbon is the low capacitance due to its limited SSA and low pack density. CNTs were initially only used directly as cathode materials for ZIHCs. Subsequently, research on CNTs as cathodes has primarily focused on combining with other active materials to form superior conductive networks within the electrode. The introduction of heteroatoms (such as N, P, and S) helps in further improving the wettability of CNTs and enhancing the adsorption kinetics of Zn^2+^ ions. In addition, CFs can be employed as conductive substrates or current collectors, which is expected to achieve higher performance of devices.

### 2D Carbon Materials

Among various multi-dimensional electrode materials, 2D materials are the most exploited. 2D layered porous carbons have demonstrated great promise in various energy-related applications due to their highly improved interaction between the components by exceptional structural design and alternation of the associated electronic properties [132, 133]. These 2D carbons with enlarged interlayer spacing can provide a large surface area and expose enriched surface atoms or electroactive sites, thereby enhancing the capacitance of carbon materials. Graphene and carbon nanosheets are the 2D carbon materials that are discussed in detail in this part.

#### Graphene-based Materials

Graphene is deemed the first carbonaceous 2D material available to us. Although 19 years have passed since the first report of it, the worldwide interest in this “miracle material” is still increasing, as evidenced by the number of publications per year and the substantial research expenditures [134]. One reason why graphene research has progressed so fast is its supreme electrical, mechanical, thermal, and transport properties [135–139]. Graphene-based materials demonstrated remarkable achievements in the field of ZIHCs owing to their exceptional electronic conductivity, large SSA, and unique pore structures [140]. However, the stacking of graphene restricts the performance of graphene-based materials. Liu et al. [141] fabricated ZIHCs with the flexible graphene@carbon cloth (graphene@CC) cathode. The graphene@CC cathode was fabricated by coating a slurry containing graphene nanoplatelet powder onto a flexible CC substrate. The graphene@CC cathode has an electricity of ∼67.1 S cm^−1^, an SSA of 678.6 m^2^ g^−1^ with numerous mesopores. Using an anti-freezing gel electrolyte membrane and a flexible Zn@CC anode, the manufactured ZIHC offers a high specific capacity of 247.7 F g^−1^, a high energy density of 158.1 Wh kg^−1^ at a power density of 236.9 W kg^−1^ and excellent long-term cycling stability of 73.7% capacitance retention after 50,000 cycles at a current load of 5 A g^−1^. Furthermore, the device demonstrated outstanding low-temperature electrochemical performances. Even at a temperature of − 20 °C, it still exhibits a high specific capacitance of 202.8 F g^−1^ at 0.2 A g^−1^, which is up to 81.9% of the initial room-temperature value. This could be attributed to the introduction of the optimum gel electrolyte membrane. Similarly, Liu et al. [142] demonstrated a ZIHC by using graphene nanosheet/carbon nanotube-intercalated nanoflaked manganese dioxide (GNS/CNT@MnO_2_) composites and Zn foil as the cathode and anode, respectively. The GNS/CNT@MnO_2_ was prepared by reacting KMnO_4_ with the GNS/CNT hybrids in ethanol. Employing a mixture solution of 2.0 M ZnSO_4_ and 0.1 M MnSO_4_ as the electrolyte, the ZIHC achieves a high capacity of 277 mAh g^−1^ at 0.2 A g^−1^. To further improve the performance of the device, the Zn anode was replaced by AC, and a pre-zincation treatment was developed to provide Zn^2+^ source. The specific energy value of the optimized capacitor GNS/ CNT@MnO_2_//AC reaches 41.5 Wh kg^−1^ at a power density of 89.08 W kg^−1^ based on the total mass of active materials in both electrodes.

**Fig. 8:**
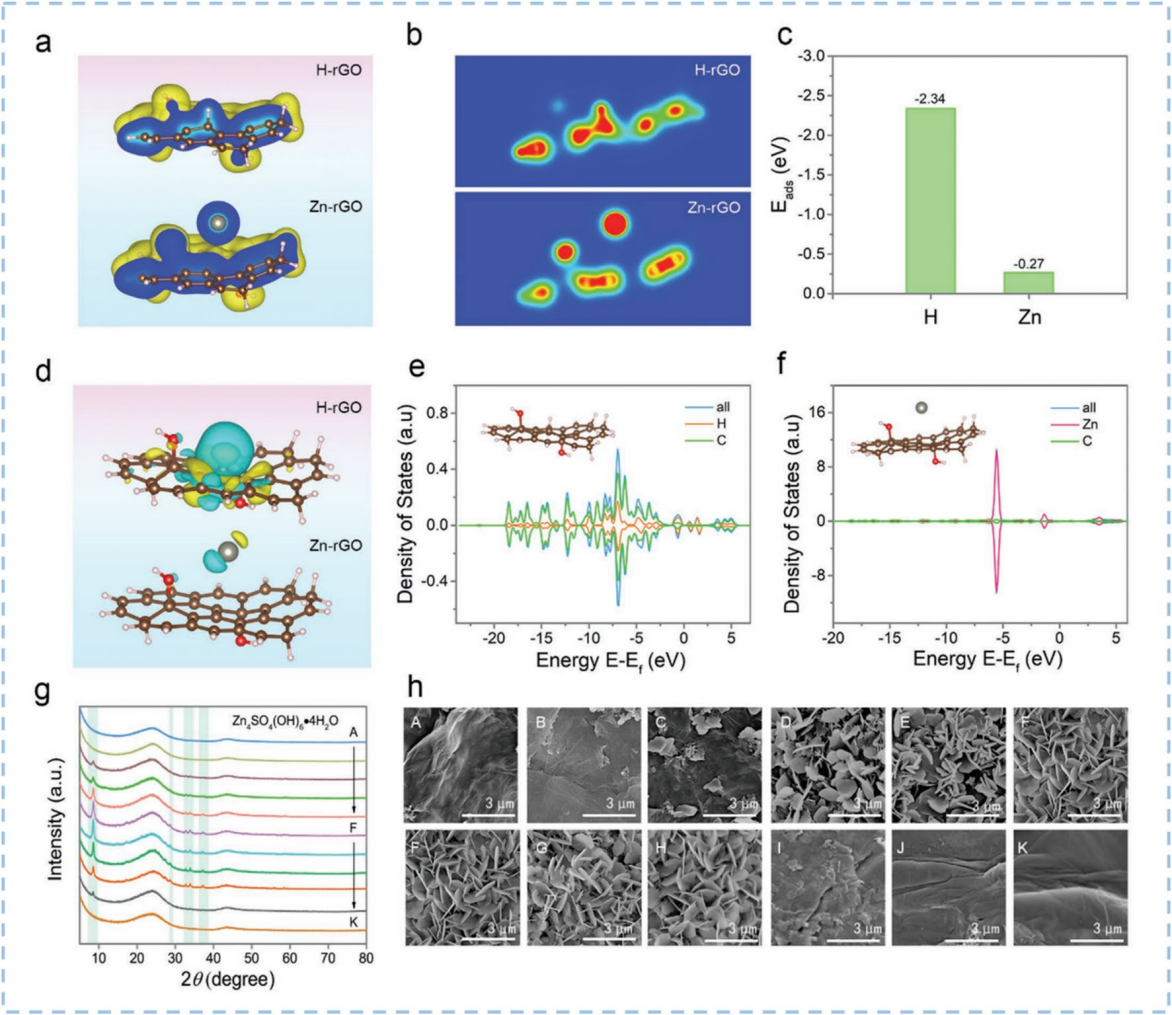
DFT calculation and ex situ SEM to verify the adsorption process of H^+^ ions on rGO. **a** The structure energy and **b** charge distribution of rGO-200 upon adsorption of H^+^ and Zn^2+^. **c** The calculated adsorption energy of H^+^ and Zn^2+^ on rGO. **d** The optimized charge-density-difference patterns and **e**, **f** density of the states of rGO model upon the adsorption of H^+^ and Zn^2+^ ions. **g**, **h** Ex situ XRD profiles and situ SEM images of rGO-200. Reproduced with permission [144]. Copyright 2022, Wiley-VCH

Reduced graphene oxide (rGO), a graphene derivative with a large surface area, has rich defects and abundant oxygen-containing functional groups. These oxygen functional groups provide additional pseudocapacitance by introducing fast redox reactions on or near the carbon surface and modifying the electrode with more hydrophilic properties, thus having a significant impact on the charge storage capacity of carbon cathodes. A significant effort is currently being dedicated to understanding the surface chemical properties and charge storage behavior of rGO. Carboxyl and carbonyl not only greatly increase the pseudocapacitive redox activity but also facilitate the chemical adsorption process of Zn^2+^ ions. Shao et al. [143] studied the oxygen functional groups of hydrogen peroxide-assisted hydrothermal rGO (HHT-rGO) cathode effects on the electrochemical charge storage capability of ZIHC. The HHT-rGO was prepared by hydrothermal reduction with the addition of hydrogen peroxide as a reducing agent. HHT-rGO demonstrates a moderate conductivity of 5.16 S m^−1^, and an oxygen content of 18.9 at%. DFT calculations prove that the carboxyl and carbonyl groups have higher Coulomb interactions with the cation Zn^2+^, which could strengthen the chemical adsorption of Zn^2+^ ions and the electrochemical charge storage. The as-prepared aqueous ZIHC based on the HHT-rGO cathode exhibits a high capacitance of 277 F g^−1^ and a long cycling durability of 20,000 cycles with 97.8% capacitance retention. In addition, the reversible adsorption/desorption behavior of H^+^ ions have been proven to occur on carbon atoms. Xu et al. [144] prepared a series of rGO samples which were annealed at different temperatures. Thus, the content of the surface functionalities of rGO could be precisely regulated. Beyond the contribution of oxygen-containing groups, H^+^ ions participate in the charge storage process by reversibly interacting with carbon atoms in the graphitic domains via adsorption and desorption, accompanied by charge transfer, C *sp*^2^–*sp*^3^ re-hybridization, and the distortion of the graphitic structure (Fig. 8). As a result, the rGO-200 sample exhibits the highest specific capacitance of 245 F g^−1^ at 0.5 A g^−1^ together with a capacitance retention of 53% at 20 A g^−1^, and remarkable cycling stability of 75% after 10,000 cycles at 10 A g^−1^ among rGO samples.

The rGO can be uniformly wrapped on the surface of the active material as a buffer layer. By combining with rGO, the structure of active materials is stabilized, and the volume change caused by the intercalation/deintercalation of Zn^2+^ ions is alleviated. Patil et al. [145] prepared a 2D layered niobium oxyphosphide nanomaterial (rGO-NbPO) through a single-step hydrothermal method. The energy storage performance of the rGO-NbPO nanosheets was superior to that of the NbPO when further utilized as the cathode in ZIHCs. The as-fabricated ZIHCs display a high specific capacitance of 191.88 F g^−1^, a good energy density of 56.03 Wh kg^−1^, and a high power density of 1000 W kg^−1^ due to the synergistic effects of the optimized electrode and electrolyte. Wang et al. [146] designed a flexible ZIHC by employing a rGO-V_2_O_5_ cathode with high electronic conductivity and exceptional structural stability. The rGO provides a flexible substrate for the electrodes, making the design of flexible electronic equipment possible. Using rGO-Mxene as the anode, the assembled flexible ZIHC displays excellent electrochemical performance with a specific capacitance of 175 F g^−1^ at a scan rate of 0.5 mV s^−1^ and an energy density of 107.2 Wh kg^−1^ at a power density of 321.6 W kg^−1^. Additionally, the electrochemical performance of the flexible ZIHC remains stable even under different bending states. A buffer layer of Zn foil can also be constructed with graphene to reduce dendrite formation and extend the lifespan of zinc anodes. Zhang et al. [147] designed a thin and uniform layer of graphene coating on the surface of the Zn anode to enhance uniform Zn electrodeposition. As revealed by the theoretical simulation, the conductive graphene layer built a stable electric field for even deposition of Zn electrode and delayed the formation of protuberances/dendrites. Additionally, the graphene reduced side reactions by blocking the electrolyte. The cyclic life of graphene@Zn anode in ZIHC reaches at least 10,000 cycles which is 200 times higher than that of Zn foil anode.

#### Porous Carbon Nanosheets

In essence, 2D porous carbon nanosheets (PCNs) with high SSA and rich micro-mesopores can provide rich interfacial active sites for ion accumulation [132, 148, 149]. There is a tradeoff between the thickness of PCNs and ion transport behavior. The optimal thickness of nanosheets can effectively prevent curling and stacking of ultrathin nanosheets, shorten the transport path, and speed up the dynamic process of ions diffusion. This renders the material with a low resistance corresponding to minimum Warburg coefficient and maximum ion diffusion coefficient for fast transport of electrolytes [150]. PCNs have been used directly as electrode materials, which is of great significance for the application of PCNs in ZIHCs. Cao et al. [151] developed a kapok-derived quasi-2D tile-shaped carbon sheet (denoted as carbon tile, CT) and single-walled carbon nanotube (SWNT) composite with a large SSA of 947 m^2^ g^−1^. The microporous of CTs provide short ion-penetration pathways and enlarge the ion-accessible SSAs for reversible ion physical/chemical adsorption/desorption. As the cathode for ZIHCs, CT/SWNTs show excellent rate and weight/area performance, reaching 114 mAh g^−1^ (1.37 mAh cm^−2^) without sacrificing volumetric capacity even at a high mass loading of 12 mg cm^−2^.

Heteroatoms can optimize the wettability and enhance the electronic conductivity of carbon nanosheets for high power output. Besides, the introduction of heteroatoms also improves the charge storage capacity of the electrode materials. Zhang et al. [152] reported a ZIHC by incorporating nitrogen and phosphorus heteroatoms into the cross-linked porous carbon nanosheets. Theoretical simulations reveal that the doping of N and P atoms can weaken the energy barrier of the reactions between the cathode and Zn^2+^ ions. The assembled aqueous ZIHC demonstrates a capacity of 103.2 mAh g^−1^ (232.2 F g^−1^) at a current density of 0.1 A g^−1^, an energy density of 81.1 Wh kg^−1^, and a power density of 13.366 kW kg^−1^.

**Fig. 9:**
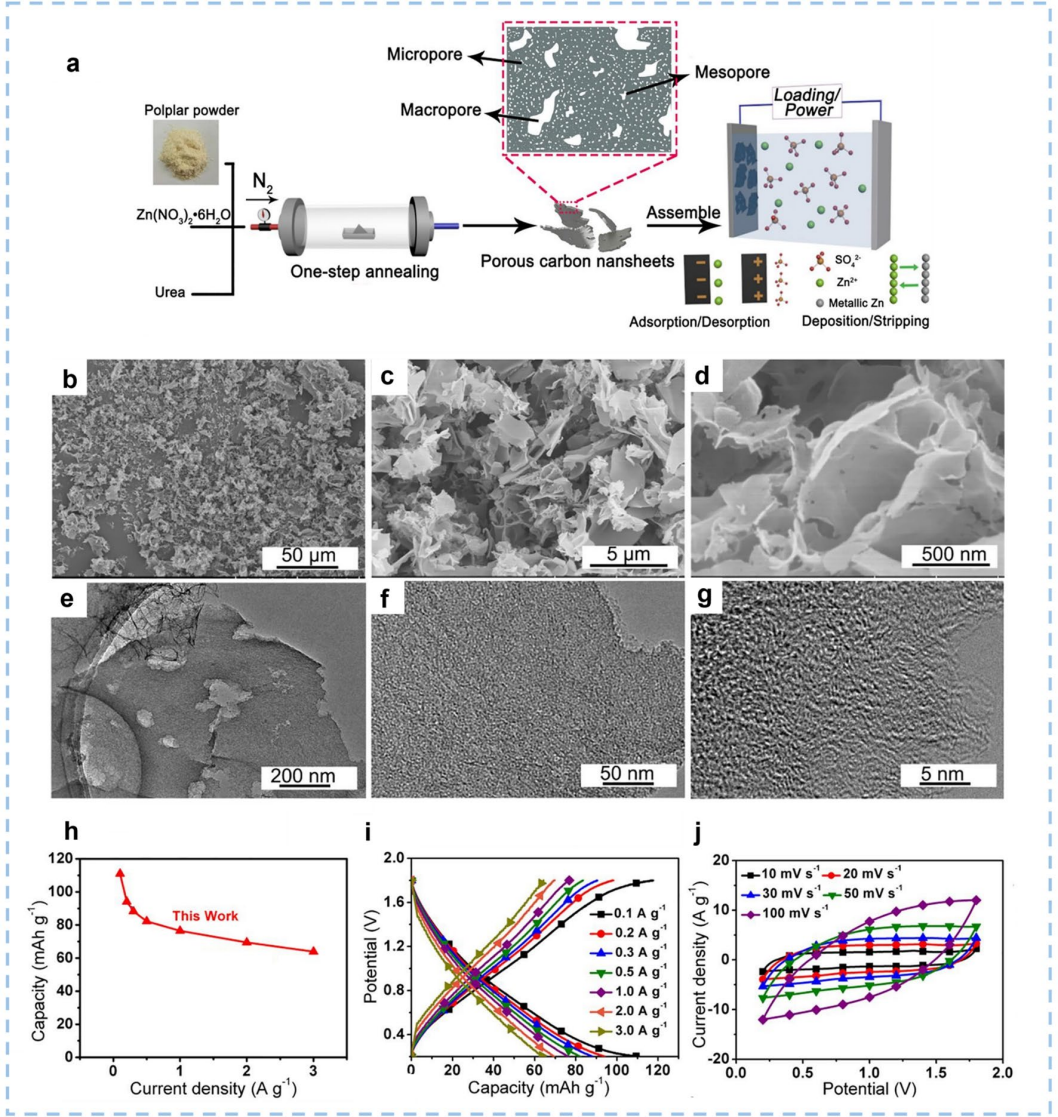
The synthesis procedure, morphology and structure of the WC-6ZnN-12U, and the electrochemical performance of the aqueous WC-6ZnN-12U//Zn system. **a** Schematic illustration for the synthesis of carbon nanosheets of WC-6ZnN-12U. **b-d** SEM images and **e–g** TEM images of the WC-6ZnN-12U. **h** Specific capacity of WC-6ZnN-12U based ZIHC. **i** GCD and **j** CV curves of the ZIHC based on WC-6ZnN-12U. Reproduced with permission [154]. Copyright 2021, Elsevier

Heteroatom-doped carbon nanosheets derived from waste biomass have been extensively investigated, while waste biomass is a cheap, readily available, eco-friendly, widely distributed, and sustainable resource. Making full use of lignocellulose biomass waste is not only a resource utilization strategy but also an important environmental conservation strategy. Biomasses, including corncob, straw, pine needles, coconut shells, bagasse, etc., can be used to prepare microporous carbon. Wang et al. [153] fabricated nanosheets functionalized with oxygen dopants and nitrogen dopants (denoted as FHPCNSs) by a two-step pyrolysis-activation strategy employing the bio-renewable aerial roots of Ficus macrocarpa as the carbon source. The FHPCNSs-800 possesses a high SSA of 1454.7 m^2^ g^−1^. Furthermore, the surface contents of oxygen and nitrogen in FHPCNSs-800 are 38.8 at% and 3.1 at%, respectively. The as-assembled FHPCNSs//Zn ZIHCs reach a high capacity of 220.1 mAh g^−1^ at 0.2 A g^−1^ and a distinguished energy density of 181.6 Wh kg^−1^ at a power density of 165.0 W kg^−1^. This research reveals that the introduction of surface pyrrolic-N and carboxyl (–COOH) functional groups cooperatively enhanced the chemical adsorption of Zn^2+^ ions. Traditional methods for synthesizing such biomass-derived 2D carbon materials involve simple pyrolysis followed by physical or chemical activation of the obtained char, but they also suffer from being corrosive to equipment and causing environmental pollution. Developing efficient and simple synthesis methods for carbon materials with superb electrochemical performance remains challenge. Lou et al. [154] fabricated N, O co-doped 2D carbon nanosheets via an innovative one-step combustion synthesis process **(**Fig. 9a). Under a nitrogen protective atmosphere, the precursor obtained by mixing poplar powder, Zn(NO_3_)_2_·6H_2_O and urea were combusted and carbonized. Then, the removal of residual zinc and compounds by washing the final product produces abundant micropores and mesopores for the resulting carbon nanosheets (Fig. 9b–g). Also, the NH_3_ gas released from the decomposition of urea and the oxygen-containing species in the original poplar served as nitrogen and oxygen dopants, respectively. The synergistic effect of N and O atoms can improve the adsorption capacity of electrolyte ions and optimize the wettability and electronic conductivity of the electrode surface, which leads to rapid charge transfer. Therefore, the ZIHC based on this N and O co-doped 2D carbon nanosheet material exhibits a superior specific capacity of 111.0 mAh g^−1^ at 0.1 A g^−1^ and an impressive energy density of 109.5 Wh kg^−1^ at 225 W kg^−1^ (Fig. 9h–j). The precise role of different nitrogen dopants still needs to be investigated.

#### Novel 2D Carbon Materials

In addition to the 2D carbon materials mentioned above, some other novel 2D carbon-based materials have also been reported. For example, Lu et al. [155] adopted a facile intercalator (H_3_BO_3_)-guided pyrolysis method to design a new type of 2D layered B/N co-doped porous carbon (LDC) using acrylonitrile copolymer (AC) as the carbon source and applied it as the cathode for ZIHCs. The optimized LDC displays a relatively high SSA of 597 m^2^ g^−1^ and a pore volume of 0.46 cm^3^ g^−1^. Microstructure analysis verified the hierarchical porosity property of the LDC. In addition, the B, N, and O doping contents are 3.0, 8.1, and 6.9 at%, respectively. The quasi-solid Zn//LDC ZIHC device exhibits intriguing Zn-storage capabilities and an exceptional energy density of 86.8 Wh kg^−1^ at a power density of 12.2 kW kg^−1^. Boruah et al. [156] fabricated the first photo-rechargeable ZIHCs using a 2D graphitic carbon nitride (g-C_3_N_4_) photo-cathode, where graphitic carbon nitride was used as the capacitor electrode and light-harvesting material. The rGO functioned as a conductive additive and selective electron transport layer in the photo-cathode, allowing the flow of photo-excited electrons to the anode through the external circuit. The photo-charging principle of the photo-ZIHCs could be explained from the energy level diagram, the photogenerated electrons were transported from g-C_3_N_4_ to FTO through rGO and finally accumulated on the Zn anode through the external circuit. Also, the photogenerated free holes attracted anions (e.g., SO_4_^2−^) towards the g-C_3_N_4_@rGO cathode for adsorption on the surface. The accumulated photogenerated electrons on the Zn anode may help the deposition of cations (Zn^2+^) from the electrolyte. The as-assembled ZIHCs show a photo-rechargeable specific capacitance of 11,377 mF g^−1^, a photo-charging voltage response of ~ 850 mV and a cyclability of 90% capacitance retention over 1000 cycles.

Overall, 2D carbon materials are ideal electrode materials for ZIHCs given their well-defined pore structure, excellent conductivity, chemical inertness, and exceptionally large theoretical SSA. The development of graphene with high utilization ratio of SSA has been receiving a special research attention in the scientific community for being electrode materials for ZIHCs. Meanwhile, a graphene coating can also shield active materials from side reactions or accommodate volume expansion, while the layered structures reduce the capacity loss caused by the dissolution of active materials in the electrolyte. Nevertheless, graphene usually results in ineffective charge storage due to restacking and agglomeration. The unique 2D structure of PCNs endows them with excellent in-plane conductivity, which can boost the in-plane transportation of electrons and the transmission of ions. Preparing PCNs with high SSA and optimal thickness to achieve outstanding capacity, accelerating the adsorption and diffusion of ions by doping heteroatoms to increase reactive sites while causing sufficient defects, and so forth are the main focuses of PCNs research currently.

### 3D Carbon Materials

When used as electrode materials, 3D carbon materials can not only provide fast electron transport paths but also significantly shorten ion diffusion length to achieve fast kinetic performance [157, 158]. Their structural interconnectivity, continuous and tunable porous structure, and good mechanical stability make them a research hotspot in various applications, particularly in the field of energy storage [40, 159–163]. The most common 3D carbon materials that can be used as the cathode of ZIHCs mainly include 3D porous carbon, 3D graphene-based carbon, and hierarchically porous carbon.

#### 3D Porous Carbon

**Fig. 10:**
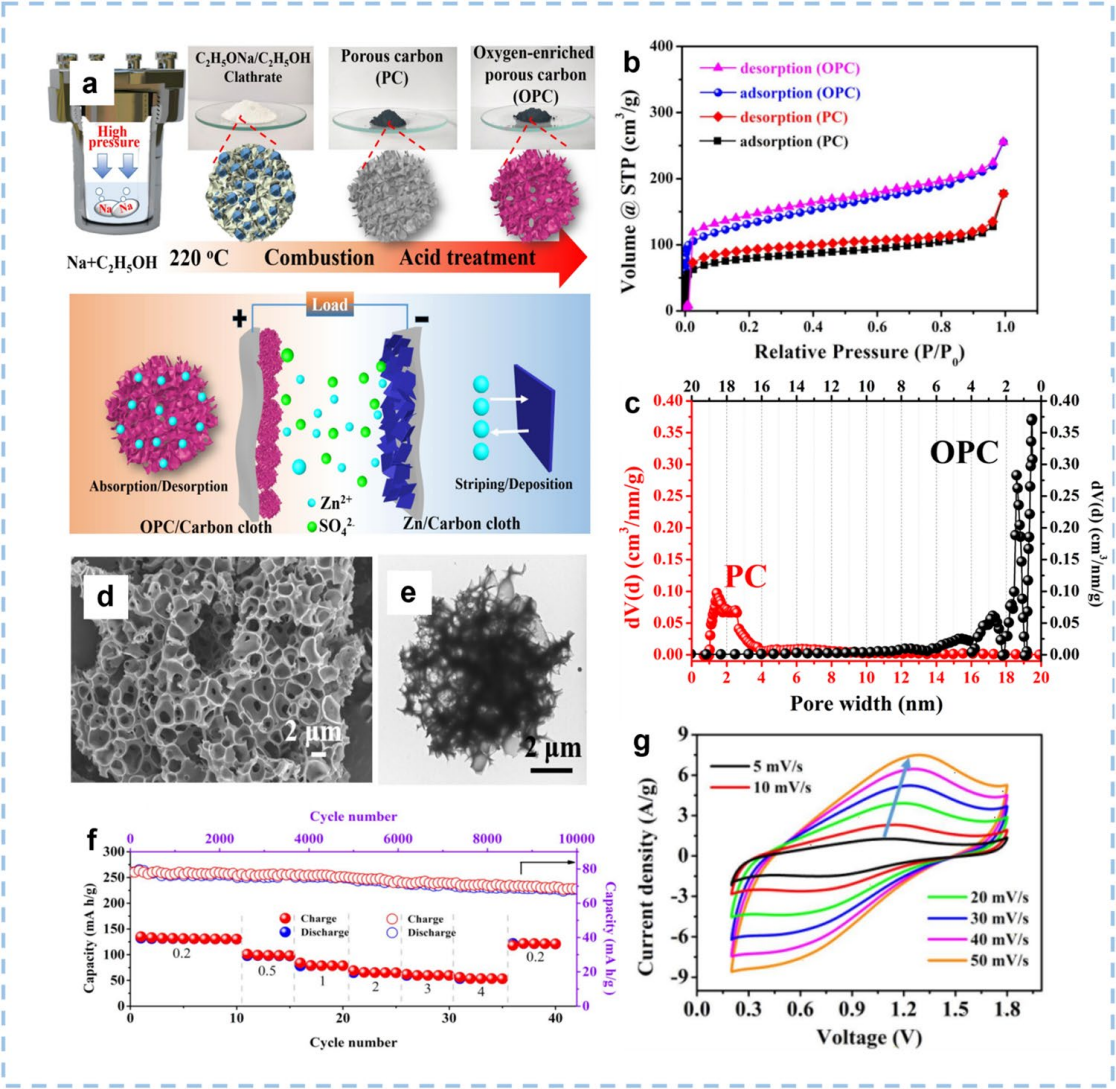
The synthesis procedure, morphology, and structure of the OPC and the electrochemical performance of the OPC-based ZIHC. **a** Schematic illustration of the stepwise preparation of OPC and the working mechanism of the assembled ZIHC based on OPC as the cathode. **b** N_2_ adsorption and desorption isotherms of OPC and PC. **c** Pore diameter distribution of OPC and PC. **d**, **e** SEM and low-magnification TEM images of OPC. **f** Rate capability at various rates and cycling stability at a current density of 1.0 A g^−1^ for OPC. **g** CV curves of PC and OPC at a scan rate of 10 mV s^−1^. Reproduced with permission [164]. Copyright 2020, Elsevier

It is challenging to design novel structures of carbon-based cathodes to afford a high diffusion speed of Zn^2+^ ions as well as a high reversible capacity, which also accords to applying these new-type cathode materials in flexible solid-state ZIHCs. Porous carbons with 3D structures has been investigated extensively in the field of electrode fabrication for high-performance ZIHCs. The transportation of ions can be facilitated by 3D porous carbon's large SSA and internally interconnected structure, which together increase the contact area with the electrolytes and shorten the diffusion paths. These properties endow the ZIHCs based on 3DPC cathodes with a high power density and exceptional rate capability. Zheng et al. [164] used a combustion approach and continuous acid treatment to obtain an oxygen-enriched 3D porous carbon (abbreviated as OPC) by employing ethanol as the carbon source (Fig. 10a). The OPC exhibits a higher SSA of 523 m^2^ g^−1^ than that of PC without acid treatment (315 m^2^ g^−1^), well-developed micropores less than 2 nm, and a higher pore volume of 0.37 m^3^ g^−1^ (Fig. 10b, c). Due to the unique pore structure of the OPC (Fig. 10d, e), fast electrochemical kinetics were endowed, and the introduced oxygen functional groups provided a prominent pseudocapacitance. A flexible ZIHC using Zn-deposited carbon cloth as the anode, OPC as the cathode, and glutaraldehyde cross-linked gelatin/ZnSO_4_ as the gel electrolyte was assembled. The flexible solid-state ZIHC achieves an excellent capacity of 132.7 mAh g^−1^, a high energy density of 82.36 Wh kg^−1^, a power density of 3760 W kg^−1^ (Fig. 10f, g), and can also be bent at different angles without obvious diminution of capacitance.

3D porous carbons derived from biomass and its byproducts have become an indispensable kind of cathode material for ZIHCs in recent years [165–170]. The 3D porous carbons derived from biomass exhibit well-developed porous structures and morphologies, as well as naturally functional groups or heteroatoms derived from the intrinsic chemical structure of these biomasses, thereby providing sufficient reaction sites and surface wettability. To achieve higher electrochemical performance, it is often necessary to add chemical activation agents to increase the SSA or optimize the pore structure of carbon materials. Wang et al. [171] prepared pine needles derived S-doped 3D porous carbon (S-3DPC) with potassium thioacetate as the activation agent. The final product obtained by carbonization at 800 °C, which was denoted as S-3DPC-800, shows an SSA of 2336.9 m^2^ g^−1^ and S-containing functional groups content of 1.71 at%. When used as cathode materials for ZHSCs, the S-3DPC-800 delivers a specific capacity of 203.3 mAh g^−1^ (123.8 mAh cm^−3^) at 0.2 A g^−1^ and 96.8% of the initial capacity retained after 18,000 cycles. Li et al. [52] synthesized a pencil-shaving-derived porous carbon, which was denoted as PSC-Ax, where x corresponds to the activation temperature. The PSC-A600 sample exhibits a BET SSA of 948 m^2^ g^−1^ and abundant micropores with sizes of 0.8 and 1.1 nm, which could provide a rich electrochemically effective area for energy storage. With 1 mol L^−1^ Zn(CF_3_SO_3_)_2_ electrolyte, the constructed aqueous ZIHC of Zn//PSC-A600 achieves a high energy density of 147.0 Wh kg^−1^ at 136.1 W kg^−1^ and a maximum power density of 15.7 kW kg^−1^ at 65.4 Wh kg^−1^ together with outstanding cycling stability of 92.2% capacity retention after 10,000 cycles at a current density of 10 A g^−1^. Notably, when employing a novel AFHE (containing PVA, Zn(CF_3_SO_3_)_2_, EG, and H_2_O), the quasi-solid-state Zn//PSC-A600 sustains about 63.9% of initial capacitance (20 °C) and ~ 100% Coulombic efficiency after 80 cycles even at − 15 °C. In another study, Lu et al. [172] demonstrated a simple route to fabricate a series of dual-templated sodium alginate-derived porous carbon (DSPCs) by KCl and self-template as dual templates. The introduction of KCl makes the DSPCs have rich 3D channels and interconnected network structures. The DSPCs-1 shows the largest BET SSA of 872.6 m^2^ g^−1^ and the highest pore volume of 0.462 cm^3^ g^−1^. Furthermore, the Zn//DSPCs^−1^ ZIHCs deliver a specific capacity of 87.5 mAh g^−1^ at 0.2 A g^−1^ and an energy/power density of 99.22 Wh kg^−1^/200 W kg^−1^.

#### 3D Graphene-Based Materials

3D graphene-based materials possess additional structural advantages beyond those of 2D graphene and 3D porous carbon. First, their bulky structure prevents the accumulation of 2D graphene sheets, preserving the properties of large SSA and exposing more active sites. Second, the well-developed pore structure can provide more ion diffusion channels, thereby shortening the distance of mass transfer [159]. Third, such a structure also makes it possible to assemble the binder-free electrode, which contributes to the much-improved conductivity of the electrode [173, 174]. Furthermore, most previous studies on the calculation of the energy density of ZIHCs are based on the mass of the active material, and there are only a small number of relevant publications that investigate the volumetric properties of ZIHCs. Zhang et al. [175] demonstrated the ZIHC employing dense high-density porous 3D graphene (DGH) as the cathode and Zn foil directly used as the anode. The DGH possesses an SSA of 208.29 m^2^ g^−1^ and a high density of 1.50 g cm^−3^, which have a positive effect on increasing the volumetric energy density of the device. As a result, the ZIHC exhibits a high specific capacitance of 222.03 F g^−1^ at a current density of 0.5 A g^−1^, a volumetric energy density of 118.42 Wh L^−1^, and a superb power density of 24.00 kW L^−1^. Furthermore, it showed excellent cycling stability with 80% capacity retention after 30,000 cycles at a current density of 10 A g^−1^.

**Fig. 11:**
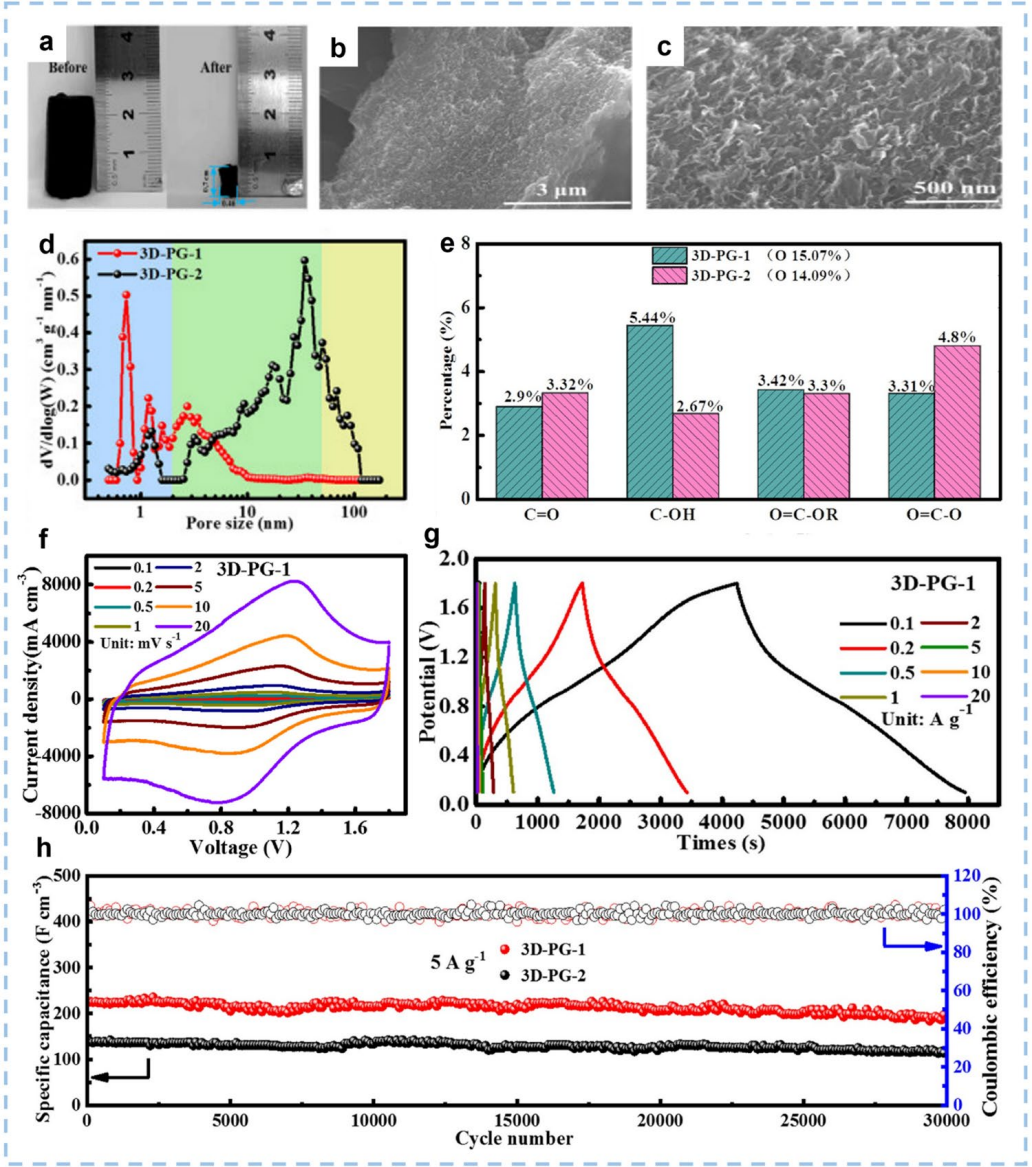
The photograph, microstructure, pore size, and the electrochemical performance of the 3D-PG-1 cathode. **a** The photograph of 3D-PG-1 (before and after different drying methods), **b** and SEM images of 3D-PG-1. **c** Magnified SEM images of 3D-PG-1. **d** Pore size distribution of 3D-PG-1 and 3D-PG-2 (the blue area is micropore, green is mesopore, and yellow is macropore). **e** The proportion of different oxygen-containing functional groups in 3D-PG-1 and 3D-PG-2. **f** CV curves at different scan rates. **g** GCD curves at different current densities. **h** Long cycling stability at 5 A g^−1^. Reproduced with permission [177]. Copyright 2022, Elsevier

Graphene hydrogel is a typical 3D graphene-based material. Since its first report in 2013, it has attracted much attention due to its excellent tensile property and especially the pseudocapacitive energy storage mechanism in the field of ZIHCs [176]. Xu et al. [177] prepared a 3D porous graphene (3D-PG-1) by drying the graphene hydrogel for a short period of capillary evaporation (Fig. 11a–c). The micropores in 3D-PG-1 are abundant, with the pore size ranging from 0.6 to 1 nm (Fig. 11d). The evaporation-induced interconnected pores (mesopores and micropores) across the 3D-PG-1 provided rich transport channels for the rapid diffusion of Zn^2+^ ions, mitigating the strong electrostatic adsorption of the Zn^2+^ on the cathode material, and enabling long-cycling stability. The abundant oxygen functional groups of 3D-PG-1 facilitate the wetting ability of the electrolyte and enhance the pseudocapacitance (Fig. 11e). Consequently, the ZIHC constructed by the 3D-PG-1 cathode and Zn anode with 3 M Zn(CF_3_SO_3_)_2_ aqueous electrolyte exhibits excellent electrochemical performance with a high specific volumetric capacitance of 299 F cm^−3^ at 0.1 A g^−1^, a good high rate capability of 150 F cm^−3^ at 20 A g^−1^, excellent long cycling stability of 225 F cm^−3^ at 5 A g^−1^ with the capacitance retention of 85% after 30,000 cycles, and an extremely high volumetric energy density up to 118 Wh L^−3^ at a high power density of 116 W L^−3^ (Fig. 11f–h).

By assembling and overlapping graphene nanosheets, a graphene aerogel with a three-dimensional cross-linked network structure is formed. The superior conductivity, hierarchical porosity, high contact area, and specific morphologies of graphene aerogel contribute to a qualitative leap in the performance and applicability of graphene aerogel-based devices [178]. Wang et al. [174] designed a 3D MXene (Ti_3_C_2_T_x_)-reduced graphene oxide aerogel with a lightweight characteristic. The MXene-rGO aerogel exhibits a porous fishing net structure with a pore size of 2–5 µm. Furthermore, when further utilized as the cathode of ZIHCs, the MXene-rGO aerogel could fully utilize the pseudocapacitance mechanism of MXene to store and deliver electrical energy. Using zinc foil as the anode, the as-fabricated ZIHC exhibits excellent electrochemical performance with a high specific capacitance of 128.6 F g^−1^ at a current density of 0.4 A g^−1^, and a high energy density of 34.9 Wh kg^−1^ at a power density of 279.9 W kg^−1^. The capacitance retention is above 95% of the initial capacitance after 75,000 charge and discharge cycles at a current density of 5 A g^−1^. It is noted that the MXene-rGO aerogels could be directly used as cathodes without binder and conductive-additive.

#### Hierarchically Porous Carbon

**Fig. 12:**
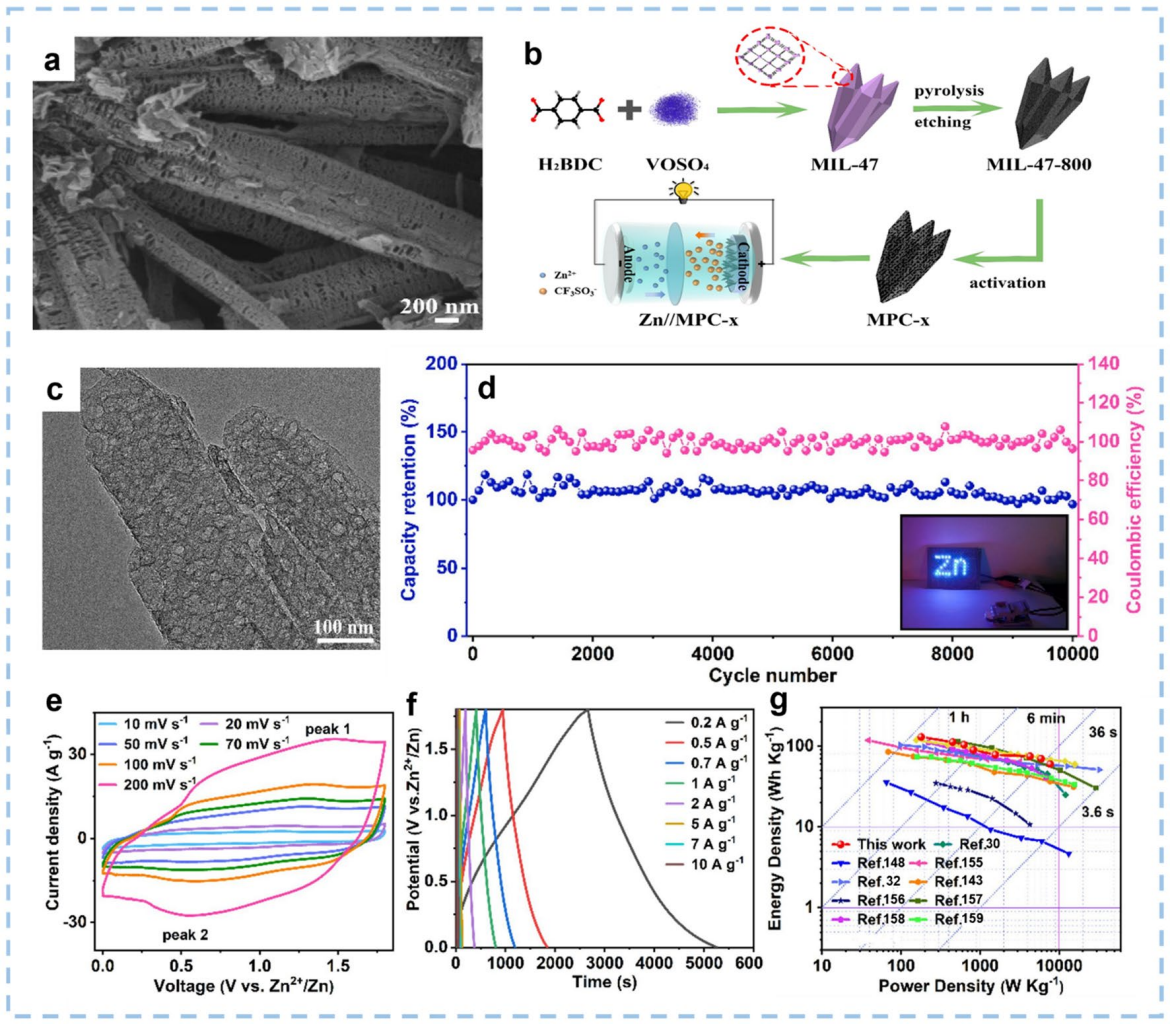
The preparation process, morphology, and structure of the MPC-2 and the electrochemical performance of Zn//MPC-2 ZIHC device. **a**, **c** SEM image and TEM image of the MPC-2. **b** Schematic illustration for the preparation of MPC and construction of the Zn//MPC-x ZIHC. **d** Cycling performance and Coulombic efficiency at 10 A g^−1^ (inset showed LED array powered by the ZIHC). **e** CV curves at different scan rates ranging from 10 to 200 mV s^−1^. **f** GCD curves at various current densities ranging from 0.2 to 10 A g^−1^. **g** Ragone plots compared with other ZIHC devices. Reproduced with permission [30, 32, 103, 154, 174, 179, 184–187]. Copyright 2022, Elsevier

The hierarchically micro/meso/macroporous structure of hierarchically porous carbon (HPC) permits rapid diffusion of Zn^2+^ ions, extraordinarily efficient electrochemically active surface area, and large capacity [179]. Micropores can increase the SSA in favor of charge accumulation, while mesopores appear as structural defects that can shorten the transport path of ions [53, 180]. For example, Chen et al. [180] prepared a 3D honeycomb-like hierarchically porous carbon (HHPCs) cathode with an SSA of 2265 m^2^ g^−1^_._ The as-fabricated ZIHC possesses a capacity of 147 mAh g^−1^ at 0.2 A g^−1^, and a power density of 40 kW kg^−1^ at 56.7 Wh kg^−1^. At present, the synthesis of HPC mainly includes the hard template method and the soft template method [181, 182]. The HPC extracted from biomass waste has the merits of easy accessibility and processibility, adjustable pore structure, and tunable surface properties. Wang et al. [169] prepared O, N-doped HPC derived from the chitin. The SSA of the obtained HPC is 1488.3 m^2^ g^−1^. Yu et al. [183] prepared an O-doped HPC derived from the oxygen-rich orange peel. The HPC possesses a large SSA of 2156 m^2^ g^−1^. In other research, Yu et al. [179] prepared a HPC cathode with a 3D interconnected structure, in which bagasse and coconut shell were used as carbon sources. The HPC possesses a high SSA of 3401 m^2^ g^−1^ and well-developed porosity characteristics. Operating under the voltage window of 0.01–1.8 V, the as-constructed ZIHCs display a high energy density of 118 Wh kg^−1^ and 94.9% capacity retention for 20,000 cycles at a current density of 2 A g^−1^. Specifically, nano porous carbon materials derived from metal organic frameworks (MOFs) with hierarchical morphology and controlled porosity have advantages for constructing ZIHCs. Since carbon materials derived from MOFs provide efficient active sites for ion adsorption and shorten the diffusion path of electrolyte ions, they exhibit superior electrochemical performance. Li et al. [184] designed a MIL-47-derived porous carbon (MPC-x) by using MOF as a precursor, where x represented the ratio of KOH activation agent (Fig. 12a–c). In detail, the MPC-2 shows an excellent conductivity of 6.3 S cm^−1^, a high SSA of 2125 m^2^ g^−1^, a hierarchically porous structure, and an oxygen-rich content of 7.6 at%. When used as the cathode material for ZIHCs, the MPC-2 delivers an impressive specific capacitance of 289.2 F g^−1^ at a current density of 0.2 A g^−1^, a high energy density of up to 130.1 Wh kg^−1^ at a power output of 180.3 W kg^−1^, and a high capacity retention of 96.7% after 10,000 cycles at a current density of 10 A g^−1^ (Fig. 12d–g).

N dopants can effectively lower the energy barrier of the chemical interaction between C-O and Zn^2+^ ions, thereby increasing the chemical adsorption of Zn^2+^ ions on the electrode surface. Zhang et al. [188] fabricated a N-doped HPC (denoted as HNPC) by thermally treating an as-prepared porous carbon (denoted as PC) precursor in an ammonia atmosphere at 850 °C. The SSA of the HNPC sample is 2762.7 m^2^ g^−1^, which is smaller than that of the PC (3314.1 m^2^ g^−1^). But the higher Zn-ion storage capacity of the HPC of 177.8 mAh g^−1^ is achieved at a high current density of 4.2 A g^−1^, while the capacitance of the PC is 67.8 mAh g^−1^. Experimental results and theoretical simulations demonstrated that N doping significantly facilitated the chemical adsorption process of Zn^2+^ ions, as well as greatly increased the conductivity, surface wettability, and active sites of the NHPC. Thus, the HNPC-based aqueous ZIHC delivers a remarkable capacity of 177.8 mAh g^−1^ under a high current density of 4.2 A g^−1^, an exceptional energy density of 107.3 Wh kg^−1^ at a power density of 24.9 kW kg^−1^ and displays admirable cycling stability with 99.7% capacity retention after 20,000 cycles. In addition to the heteroatom mono-doping strategy, co-doping with other heteroatoms can obtain a higher capacitance. Zhang et al. [54] produced the boron (B) and sulfur (S) co-doped spongy-like HPC (B_2_S_3_C) via a one-pot doping calcination process using K_2_B_4_O_7_·4H_2_O and C_2_H_3_KOS as boron and sulfur sources. The B_2_S_3_C possesses a large SSA of 2364 m^2^ g^−1^, which can provide rich active sites for ion transport. The contents of O, B, and S atomic contents in B_2_S_3_C are 0.91% and 1.38%. The experimental results and DFT calculations demonstrate that the B_2_S_3_C co-doped with B and S atoms have good surface wettability, electronic conductivity, abundant active sites, and sufficient charge storage space. The assembled ZIHC based on the B_2_S_3_C exhibits a high specific capacity of 182.6 mAh g^−1^ at 0.1 A g^−1^, a remarkable energy density of 292.2 Wh kg^−1^ at a power density of 62.2 W kg^−1^, and a capacity retention of 96.2% after 10,000 cycles. The above two studies highlight the relationship between the heteroatom co-doping strategy and tuning the performance and functionality of devices. Shang et al. [55] developed a hierarchically porous carbon sphere (HPCS) co-doped with N, P, and S heteroatoms by the heterogeneous nucleation and SiO_2_ template methods. The HPCS-900 sample has a large SSA of 1176.95 m^2^ g^−1^ and the contents of N, P, and S in the HPCS-900 are 3.16, 2.3, and 1.45%, respectively. Operating within the voltage window of 0.1–1.7 V, the ZIHC with HPCS-900 as the cathode enables a capacity of 104.7 mAh g^−1^, energy/power density of 90.17 Wh kg^−1^/81.2 W kg^−1^, and a capacity retention of 95.24% up to 30,000 cycles.

The use of the HPC cathode with oxygen functional groups can also significantly improve the electrochemical performance of devices. Wang et al. [189] constructed functionalized HPC materials by direct pyrolysis of potassium citrate at different temperatures. The obtained materials were denoted as HPC-x (x represents the pyrolysis temperature). The SSA value for the HPC-600 is 1259.7 m^2^ g^−1^. ZIHCs based on the HPC-600 demonstrate a specific capacity as high as 169.4 mAh g^−1^ at a current density of 0.1 A g^−1^ and a specific energy density of 125.1 Wh kg^−1^. Even at a high specific current of 20 A g^−1^, the HPC-600 can achieve a high specific capacity and power density of 97.6 mAh g^−1^ and 16.1 kW kg^−1^, respectively. This may be distributed to the hydroxyl group of the HPC, which can effectively boost the chemical adsorption capability of Zn^2+^ ions. Due to the formation of the specific C–O–Zn structure, hydroxyl oxygen can adsorb Zn^2+^ ions, thereby introducing additional pseudocapacitance. The high content of hydroxyl groups can supply abundant interaction sites for Zn^2+^ ions and further enhance the storage capability of HPC-600 for Zn^2+^ ions. Carboxyl groups interact with Zn^2+^ ions mainly to form C–OO–Zn bonds, and carbonyl groups can significantly enhance the wettability of aqueous electrolytes.

In brief, 3D carbons with internally interconnected structures and well-developed porous morphologies can offer superior ion transport property and achieve superior ion storage capability in contrast to 0D and 2D carbons. 3D porous carbon was developed as an electrode for ZIHCs, demonstrating high power density and exceptional rate capability. Still, the high rate capability could be optimized by the rational design of 3D structure. ZIHCs with high rate capability could be suitable for some specific applications. Graphene hydrogel and graphene aerogel are subsets of 3D graphene materials. Their bulky structure and well-developed interior pores offer more electrochemical active sites and accelerate mass transfer. The HPC is advantageous in its hierarchical pore network, large SSA, excellent conductivity, and homogeneous pore size distribution. Recent research on HPC can be commonly divided into several categories: (1) Improve the preparation method of HPC to optimize the structure, which enhances its electrochemical performance at high current densities. (2) Prepare composite materials, introduce heteroatoms, or optimize the pore architecture with multi-dimension to improve their capacity performances.

## Current Collectors and Separators

### Current Collectors

In a typical ZIHC, when Zn metal is used as the anode, the role of Zn^2+^ is to shuttle between the electrolyte and the electrodes to maintain a high level of energy density. As an inactive part of the ZIHC, current collectors do not contribute charge storage. Their main function is to carry active materials, collect current, and conduct electrons to the external circuit [190]. During the charge and discharge processes, side reactions of the inorganic salt in the aqueous electrolyte would corrode the current collector. Thus, for ZIHCs, current collectors with strong oxidation resistance and excellent electrolyte corrosion resistance are particularly required.

In most reported works, most ZIHCs use current collectors made of stainless steel and Ti foils/foams, as shown in Table 1. The components of stainless steel (iron, nickel, chromium, and molybdenum) exist as the most thermodynamically stable oxides in solutions with 5.4 pH and 6.6 pH. Because of this aspect, passive oxide films can form on the stainless steel current collectors [191]. As for the Ti foils or foams, due to their chemical inertness, the stable TiO_2_ layer on the surface will protect them from further oxidation. However, these inert layers have high resistance, which would impede electron transportation. Additionally, Yu et al. [192] analyzed the electrochemical stability of representative current collectors in aqueous electrolytes. The OER/HER process of electrolytes on the Ti mesh needs to be triggered at a relatively high positive or low negative potential. So, Ti mesh has the advantage of working well even when the potential is relatively high. However, the high mass density of stainless steel and the high cost of Ti foils or foams are the issues that need to be solved.

It is possible to fabricate ZIHCs using carbon-based current collectors because of their excellent conductivity, high flexibility, tensile strength, and modifiable interface. Carbon-based current collectors such as CC, CNTs, graphite paper, etc., are commonly used. Huang et al. [193] prepared a PCC current collector by air calcination treatment. Compared with pristine CC, the PPC with an air calcination process has abundant oxygen-containing groups and a larger SSA. All these properties give the PCC an enhancement of the absorption of Zn^2+^ ions, which is beneficial to the loading of active materials. Li et al. [43] assembled ZIHCs by adopting graphite paper as the current collector, accompanied by dual-doped carbon hollow nanospheres as the cathode. As a result, the as-fabricated ZIHC delivers an exceptional energy density of 116.0 Wh kg^−1^ at 141 W kg^−1^, a superior power density of 21,660 W kg^−1^ at 36.1 Wh kg^−1^, as well as ultralong cycling stability up to 12,000 cycles. Carbon collectors remain impractical for practical applications due to their high thickness and fragile nature for manufacturing.

**Fig. 13:**
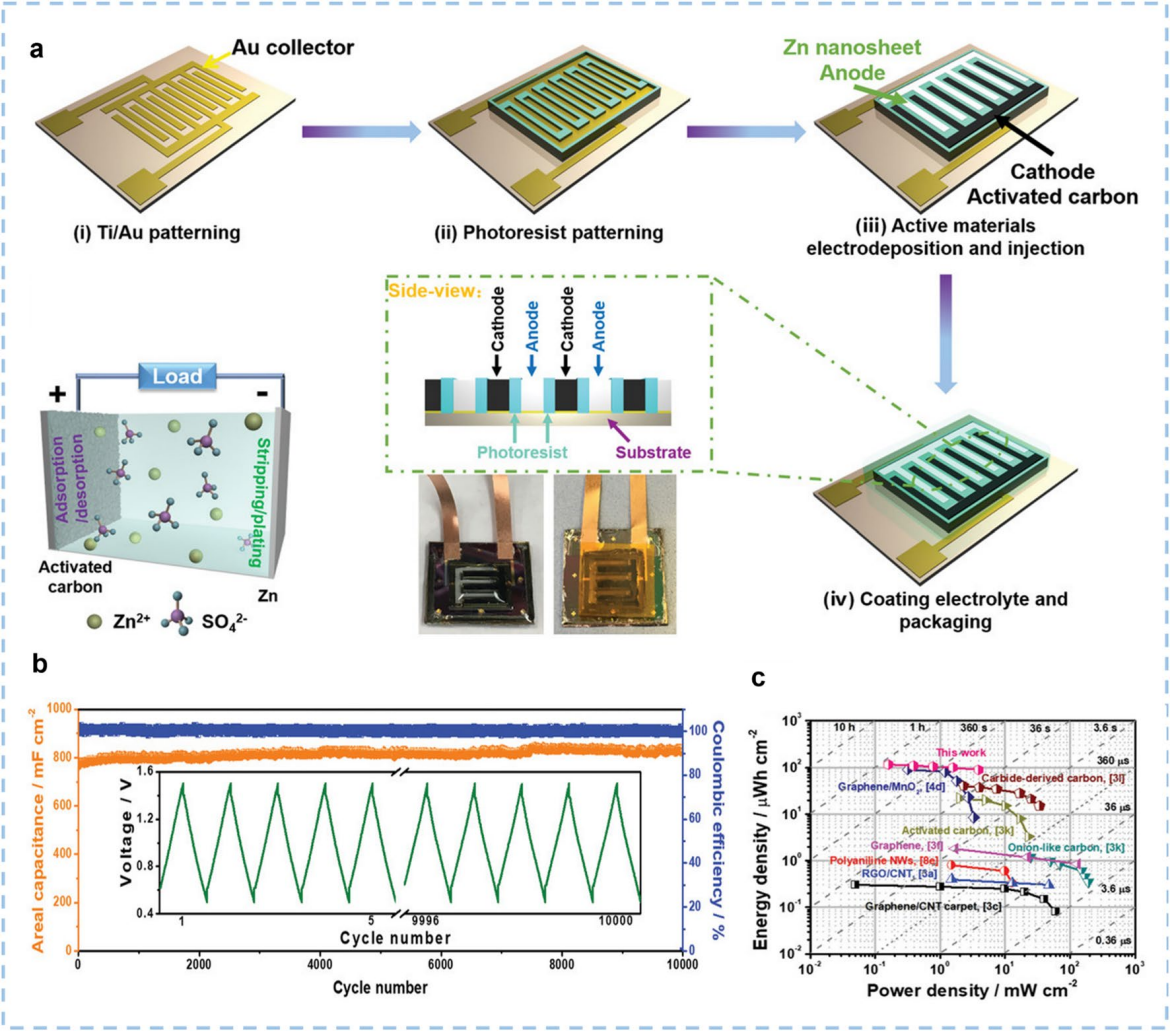
Fabrication process and the electrochemical behavior of the Zn-ion hybrid MSCs. **a** Schematic of the fabrication process of Zn-ion hybrid MSCs **b** Cycling stability at the current density of 1.56 mA cm^−2^. Inset shows the first five and the last five GCD curves of Zn-ion hybrid MSCs. **c** Ragone plots for Zn-ion hybrid MSCs, carbon-based MSCs, graphene-based MSCs, and conducting-polymer-based MSCs. Reproduced with permission [15]. Copyright 2018, Wiley-VCH

Novel effective current collectors for ZIHCs have emerged. Zhang et al. [15] assembled Zn-ion hybrid micro-supercapacitors (MSCs) which used the electrodeposited Zn nanosheets at a constant current density on the gold interdigital finger and used them as the anode (Fig. 13a). The as-fabricated Zn-ion hybrid MSCs exhibit an areal capacitance of 1297 mF cm^−2^ at 0.16 mA cm^−2^ (259.4 F g^−1^ at a current density of 0.05 A g^−1^), an areal energy density of 115.4 µWh cm^−2^ at 0.16 mW cm^−2^ (Fig. 13b, c).

The research attention on improving the energy density of ZIHCs is mainly focused on optimizing and developing electrode materials, or directly increasing the proportion of active materials in electrodes. However, current collectors, which mainly serve as conductive carriers of electrons, are often neglected. It is believed that using lightweight current collectors is a keyway to achieve higher energy densities in ZIHCs. Additionally, as an ideal current collector for ZIHCs, the following requirements must be met: (i) Good conductivity, which is beneficial to the transportation of electrons; (ii) Chemical properties of the current collector are stable, and it does not react with active materials, binders, and electrolytes; (iii) Good compatibility and high bonding strength with active materials, binders, etc., and (iv) Cost-effectiveness, and easy accessibility to large-scale production. While challenges remain in developing ideal current collectors, especially in terms of manufacturability, the potential and motivation for improvement are clear.

### Separators

A separator is situated between the anode and cathode. It plays a vital role in minimizing internal resistance while maintaining proper electronic insulation [194]. As of now, dendrite growth, surface passivation, and by-product formation are the main bottlenecks of Zn anodes [195]. The formation of zinc dendrites is a multi-step process that involves uneven zinc-ion distribution, zinc nucleation, crystal nucleus growth, and further deposition steps. In addition, once formed, dendrites cannot be eliminated entirely through dissolution during continuous cycling. Zinc electrodeposition behavior is determined by the microstructure of the separator. GF is the most common separator in the lab-scale investigation of ZIHCs, as shown in Table 1. During zinc electroplating, glass fiber (GF) and polypropylene porous separators promote zinc deposition to fill the separator pores. Moreover, GF separators are easily penetrated by zinc dendrites due to their fragile nature, large uneven pores, ans their low mechanical strength [49, 196–198]. This undoubtedly increases the risk of short circuits and leaves behind “dead zinc” when the zinc is stripped [194, 199].

**Fig. 14:**
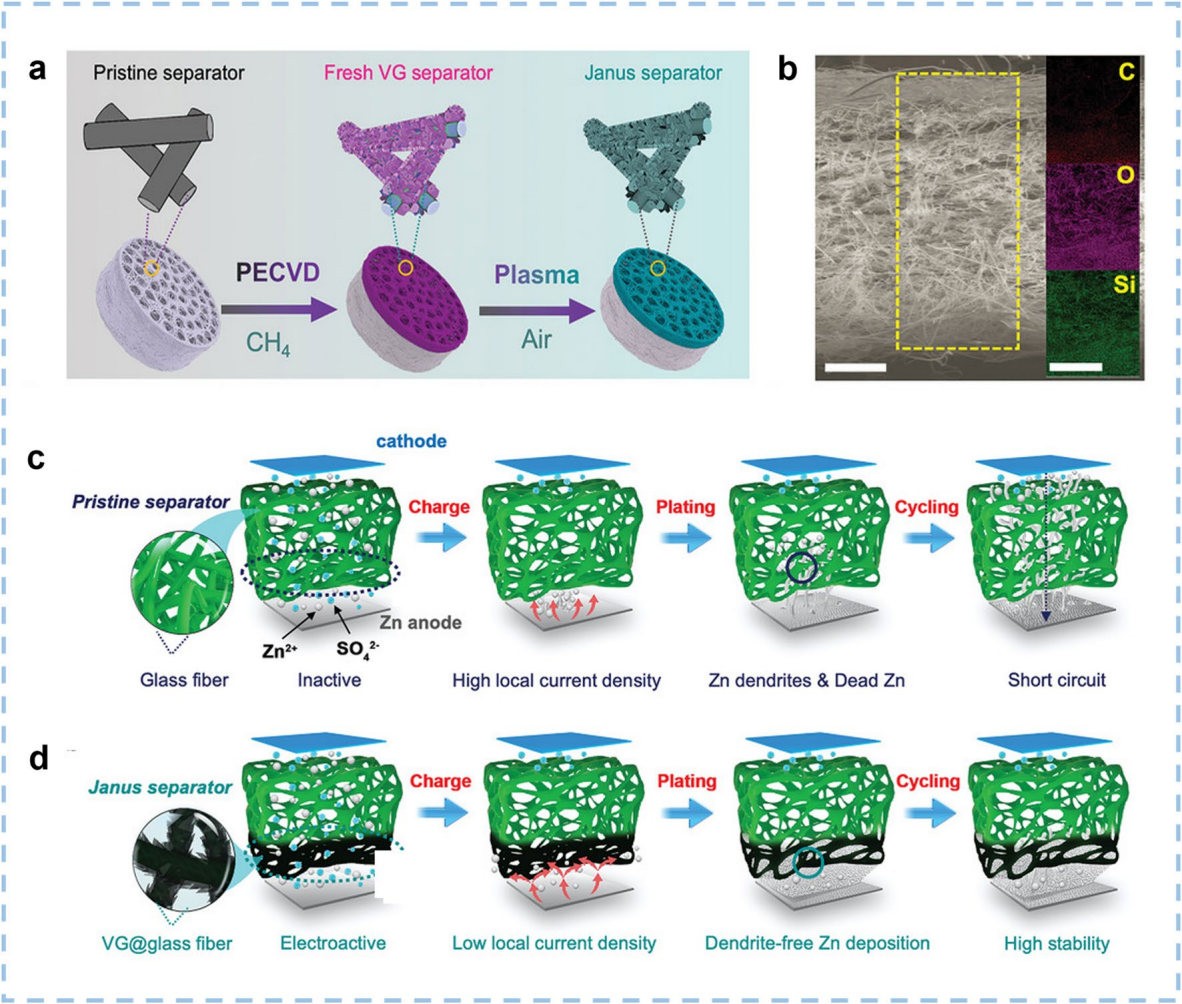
Schematics of the preparation process, morphology, and structure of the Janus separator, and the design of the Janus separator targeting stabilized Zn anode. **a** Schematic representation of synthetic process of Janus separator. **b** Cross-section SEM image of Janus separator and corresponding EDS maps. **c** A pristine glass fiber separator, and **d** a Janus separator harnessing one-side directly grown VG carpet to help lower local current density and homogenizing ion distribution. Reproduced with permission [197]. Copyright 2020, Wiley-VCH

Designing flexible separators with zinc-philic groups to guide uniform zinc deposition is a viable strategy. Li et al. [197] designed a Janus membrane by directly growing the vertical graphene (VG) carpet on the side of the GF membrane by plasma enhanced chemical vapor deposition (PECVD) strategy (Fig. 14a). Compared with pristine separator, this 3D VG scaffold has a sufficient surface area, a porous structure, and a zinc-philic feature. Further, the 3D VG conductive networks facing Zn anode could effectively homogenize electric field distribution and reduces the local current density at the side of Zn anode, thereby ensuring uniform zinc plating/stripping and maintaining high reversibility (Fig. 14b–d). As a result, the Janus separator is effective in V_2_O_5_//Zn batteries (75% capacity retention after 1000 cycle) and AC//Zn hybrid capacitors (70 mAh g^−1^ at 5 A g^−1^, 93.7% capacity retention after 5000 cycles). MXene membranes have been repeatedly shown to be effective in regulating zinc deposition behavior [200–202]. By spraying and printing MXene nanoplates on one side of the commercial GF, Su et al. [203] designed a scalable Ti_3_C_2_T_x_ MXene-modified Janus separator. For homogenizing local current distribution and promoting Zn nucleation kinetics, the MXene-GF separator developed in this way had an abundance of surface polar groups, as well as good electrolyte wettability, and high ionic conductivity. Notably, MXene-GF shows a tunable dielectric constant (ε) with an optimized value of 53.5. Due to the ε difference, the Maxwell–Wagner effect can be used to polarize the GF, which can establish a built-in electric field with 50% enhanced intensity to speed up the dynamics of mass transfer. Therefore, the performance of the zinc anode was stabilized, which provided a uniform interfacial ion field. Other types of surface-modified separators, such as Janus separators with MOF/rGO functional interlayers [204], MXene@NiO modified separators [205], BTO-decorated fiber [206], metallic Sn-coated separators [207], GF@SM separator [208], have also been reported.

Finding a new separator to replace GF is of great significance for the development of ZIHCs. For example, the Nafion film can homogenize the electrical field and Zn^2+^ concentration field on the anode surface owing to the Donnan potential. Using the Nafion separators, the growth of zinc dendrites can be restricted, and the cycle life of zinc anodes is obviously prolonged [209]. However, the prohibitive cost of the Nafion separators limits their large-scale application. Cellulose is the most prevalent natural polymer. Cellulose-based separators with a high concentration of hydroxyl functional groups have uniform and dense nanopores, as well as greater strength and modulus, and higher ionic conductivity (permeated with electrolyte). They can promote the migration of Zn^2+^ ions, lower the desolvation energy barrier of hydrated Zn^2+^ ions, reduce the nucleation overpotential of zinc deposition, and accelerate the kinetics of zinc deposition at the zinc electrode/electrolyte interface because of these merits. Zinc dendrites and potentially harmful side reactions can thus be effectively suppressed by the cellulose separator [198]. Cao et al. [210] reported a cellulose/GO composite separator (CG). After passing through a cellulose/graphene oxide (CG) separator, Zn^2+^ ions exhibit regular hexagonal deposition with a preferred crystal orientation ((002) plane) on the zinc anode. The Zn anode with CG separator exhibits lower overpotential, higher Coulombic efficiency (98.69%), and longer cycling performance (> 1750 h). Moreover, separators for lithium-ion batteries and lithium-sulfur batteries have stimulated the development of poly(acrylonitrile) (PAN) polymers for aqueous zinc ion batteries [211]. It is the –CN group in PAN that keeps Zn^2+^ ions from nucleating and growing out of control by homogenizing the electric field distribution. In short, the importance of separators should be self-evident. Suppression of zinc dendrites is often achieved by adjusting electrode structure and developing new electrolytes [212–215], but the significant role of the separator is often overlooked. Consequently, new ways to functionally modulate zinc dendrites through exploring new types of separators should be pursued.

## Summary and Outlook

In summary, benefiting from the low cost, high SSA, ordered porosity, and excellent chemical stability, carbon materials with different dimensions have shown great opportunities for advanced electrodes of ZIHCs. The reason why carbon materials are successfully widely used in ZIHCs is because of the following key properties: First, the properties of carbon materials, such as excellent electrical conductivity, excellent electrochemical activity, adjustable microstructure and surface chemistry, low cost, environmental friendliness, and ease of synthesis (the main synthesis methods for carbons are summarized in Table 2). Second, carbon materials can be stable in 0D to 3D, and carbon materials with different dimensions have different microscopic forms and functions. When used as electrode materials, their high SSA and porosity can provide high conductivity and rich transfer channels of electrolyte ions. This is a necessary factor for carbon materials to obtain high specific capacitance. Third, carbon materials have good compatibility with other materials, such as carbon materials and pseudocapacitance materials, so as to overcome the inherent defects of some materials. Take the composite of V_2_O_5_ and CFs as an example, CFs can not only avoid the agglomeration of V_2_O_5_ nanosheets and buffer the volume expansion during the charging and discharging process, but also enhance the electrical conductivity.

**Table 2:** A summary of Advantages and disadvantages of synthesis methods for carbons

Synthesis method	Advantages	Disadvantages	Practical application
Hydrothermal method	Simple to operate Mild experimental conditions Easy to surface modification and element doping	Small SSA Products with underdeveloped pores	CSs, CNF, graphene, rGO
Chemical vapor deposition method	Cost-friendly High yield Easy control of experimental conditions	Poor controllability of morphology	CSs, CNT, CNF, PCNS
Physical activation method	Simpe production process Does not corrode equipment	Relatively weak gas activator reactivity Pore structure in carbon precursor that is difficult to form	3DPC, HPC
Chemical activation method	High yield High SSA	Corrosion of equipment Environmental pollution	CNT, PCNs, 3DPC, HPC
Hard-Template method	Large SSA Adjustable morphology and pore structure	High cost Complicated preparation process	CSs, HBC, HPC
Soft-template method	Easy to synthesize Controllable synthesis conditions	Difficult to control the pore structure precisely	CSs

Carbon materials, however, still have some inherent shortcomings. Research on how to effectively improve the electrochemical performance of carbon materials has also been going on. Heteroatoms doping or surface functionalization significantly facilitated the electrical conductivity, surface wettability, and the chemical adsorption capability of Zn^2+^ ions, which leads to outstanding electrochemical performance of carbons. For the single-atom doping strategy, most of the doping atoms reported in the literature are N, B, S, P, F, Se. Multi-atom co-doping can make a synergistic effect between heteroatoms with different electronegativity and atomic size, but how to accurately regulate the doping amount of heteroatoms atoms still needs to be explored. Besides, carbon materials are used as substrates and compounded with pseudocapacitance materials to obtain materials with composite structure. The introduction of pseudocapacitance materials can improve the power density and energy density, and in turn, carbon materials can enhance the electrical conductivity. Dimensionality upgrade, that is, the transformation from low-dimensional materials to high-dimensional materials is realized by assembling carbon structural units. This can lead to carbon materials with well-developed pore structure, larger SSA, and higher mass transfer rates. For example, 2D graphene usually suffers from ineffective charge storage due to their restacking and agglomeration. By self-assembly 2D graphene, the connection of graphene is realized. The prepared bulk materials form ice crystals in the freezing process, then the ice crystals are removed by freeze drying, and pores are formed, and finally 3D graphene is obtained. The bulky structure and extra-large porosity, and well-developed interior pores of 3D graphene offer more electrochemical active sites and significantly accelerate mass transfer.

**Fig. 15:**
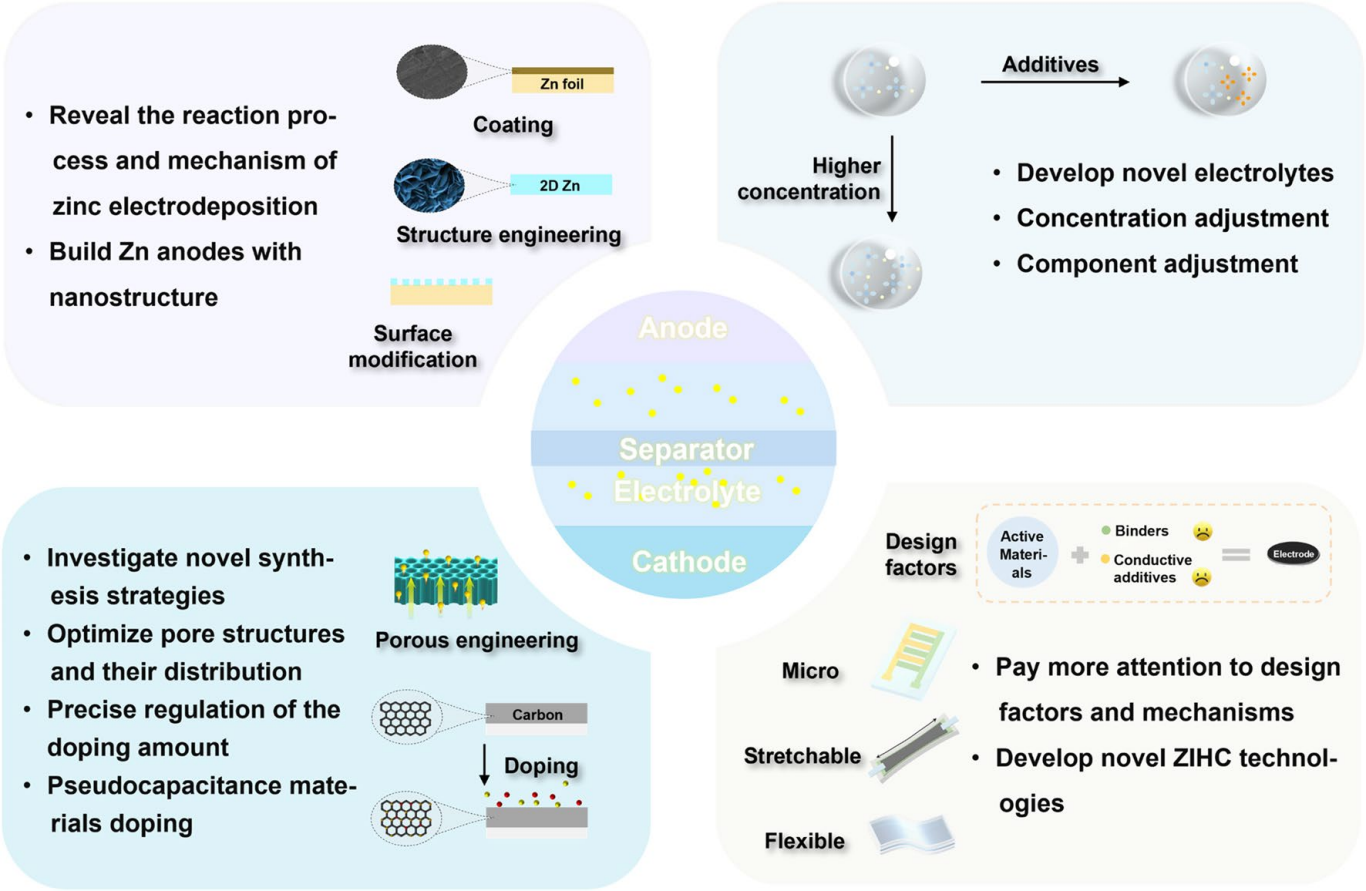
Design strategies and perspectives of Zn anodes, carbon-based cathodes, electrolytes, and ZIHC devices

On this basis, the recent research progress in different dimensional carbon materials in the pursuit of high-performance ZIHCs is briefly summarized, focusing on their morphologies, structures, and electrochemical properties. The status quo of current collectors and separators used in ZIHCs is also briefly introduced. Likewise, the investigation of ZIHCs is in its infancy. Compared to traditional secondary batteries and supercapacitors, their relatively low specific capacitance and energy density are the bottlenecks at this stage. In our view, the future strategies for the ZIHC field include the following (Fig. 15):

1. Design carbon materials that meet the performance requirements, and deeply explore the modification strategies. An ideal electrode material should meet the basic requirements of large SSA, high electrical conductivity, suitable pore structures and good wettability. For this reason, it is particularly important to rationally design the structure of carbon materials, which is also the best choice to optimize and improve the capacitance performance of carbon cathode materials. It is better to combine carbon materials with different dimensions to exert the synergistic effect of carbon materials with different dimensions than to use carbon materials with single dimension. So far, researchers have designed many hybrid structures based on different dimensions of CF integration. More efforts should be put into designing other carbon materials with composite dimensions in the future. Furthermore, it is necessary to investigate novel synthesis strategies for porous carbons with excellent compatibility between pores and electrolyte ions. Heteroatom doping of the carbon matrix is a common strategy for achieving higher capacitance of carbon materials, but the way to achieve precise regulation of the doping amount is still poorly understood. Any improvement in the properties of the carbon materials, such as wettability, structural stability, surface chemistry, and so on, after doping heteroatoms should also be considered.
2. Design new current collectors and separators that can be used on large scale. As an indispensable part of the components of ZIHCs, the role of current collectors and separators cannot be ignored. The stainless steel mesh, Ti foils/foams, and carbon-based current collectors are commonly used, however, the high mass density of stainless steel, the high cost of Ti foils or foams are the issues that restrict their practical applications. Developing lightweight current collectors or current collector-free electrodes can free up more space for active materials and help to increase the energy density of devices. This is a matter of concern. As for the separators, they can effectively isolate the contact between the anode and cathode and prevent short circuit. However, after repeated cyclic charging and discharging processes of ZIHCs, the formation of zinc dendrites may inevitably pierce the separators, which seriously affects the performance of the device. New ways to functionally modulate zinc dendrites through exploring new types of separators should be pursued. Moreover, from the perspective of practical application, factors such as cost, and process selection should also be considered.
3. Develop novel ZIHC fabrication technologies and pay attention to design factors. Flexible devices that can bend and stretch have become widely used in our daily life in recent years. Flexible ZIHCs are members of the family of flexible devices and hold vast potential for future development. Besides the electrode materials themselves, the excellent performance of a device is closely related to the design factors of the electrode and device. Currently reported carbon cathodes are composed of active materials, conductive additives, binders, and current collectors. The existence of the latter three components leads to lower gravimetric capacity and energy density of ZIHCs. Thus, extrinsic parameters such as the use of conductive additives, pre-conditioning electrodes, the relatively complicated synthesis process, and the selection of ideal separators should be evaluated.
4. Improve the temperature adaptability of ZIHCs. The use of aqueous electrolytes makes ZIHCs safer than other energy storage systems employing organic electrolytes. However, the freezing of aqueous electrolytes typically results in a dramatic loss of ionic conduction, seriously restricting the low-temperature application of such ZIHC devices. To solve this issue, scientists typically add additives to the electrolyte, such as organic solvents or additional solutes.
  In most cases, the limited working voltage of an aqueous electrolyte leads to low energy density and unstable electrochemical performance of devices. It is feasible to use high-concentration aqueous electrolytes under laboratory conditions, but in view of actual factors, it will lead to a significantly increased cost. Research on developing various electrolytes, such as salty ice, hybrid electrolytes, solid-state electrolytes, and ionic liquid electrolytes, has been going on all the time, but no ideal electrolyte that simultaneously achieves conductivity, low viscosity, a wide voltage window, a wide working temperature range, and excellent stability has been discovered. More efforts should be devoted to the study of the mechanisms and of ZIHCs. The existing Zn anodes are constrained by issues such as dendrite formation, low coulombic efficiency, short cycle lifetime, and poor rapid charge–discharge ability. At present, the improvement of Zn anodes includes structure design, surface modification, and coating to enhance the corrosion resistance. Attention should be paid to the development of Zn anodes with three-dimensional structures, which is an effective method to solve the above problems. Furthermore, revealing the reaction process and mechanism of zinc electrodeposition cannot be ignored.

All in all, with the explosive increase of demand for electrochemical energy storage, ZIHC have drawn the attention of researchers worldwide. The progress and exploration in the field of zinc ion capacitors have never stopped. Up to now, people have developed printable micro-ZIHCs [216], stretchable ZIHCs [217], anti-freezing ZIHCs [141] as well as edible and nutritive micro-ZIHCs [218]. It is believed that there will be many new types of ZIHC in the future. There is still a long way to go but this is a highway.
